# Supplementary material for: Primary Antibiotic Resistance of Helicobacter pylori in Different Regions of China: A Systematic Review and Meta-Analysis
Source: Pathogens. 2022 Jul 12;11(7):786. doi: 10.3390/pathogens11070786 (PMC9316315; doi:10.3390/pathogens11070786)
Supplement: Supplementary file 1 [file pathogens-11-00786-s001.zip › pathogens-1745230-supplementary.pdf]

Table S1 Characteristics of the enrolled studies on resistance rate of *H. pylori* to antibiotics based on Gender

Authors	Regions	Year	Sex	Method	Clarithromycin		Metronidazole		Levofloxacin	
					Patients	Prevalence	Patients	Prevalence	Patients	Prevalence
Zhang [58]	Guangdong	2000	Male				48	52.75		
			Female				37	50.68		
Hu [71]	Yunnan	2000-2001	Male				11	50.00		
			Female				10	90.91		
Gu [31]	Shanghai	2005-2006	Male				6	40.00		
			Female				10	47.62		
Lin [32]	Shanghai	2008-2009	Male		19	20.88	67	73.63	28	30.77
			Female		16	28.07	50	87.72	21	36.84
Tan [35]	Shanghai	2009-2010	Male		21	35.00	47	78.33	27	45.00
			Female		23	38.33	52	86.67	23	19.17
Liu [12]	Beijing	2012-2013	Male		30	37.97				
			Female		19	37.25			-	-
Zhang [11]	Beijing	2009-2010	Male		312	46.57	398	59.40	292	43.58
			Female		336	51.61	452	69.43	357	54.84
Zhang [14]	Beijing	2013-2014	Male		164	46.72	209	59.54	174	49.57
			Female		187	53.58	238	68.19	207	59.31
Hu [39]	Shanghai	2013-2015	Male		8	11.43	45	64.29	16	22.86
			Female		11	17.74	40	64.52	24	38.71
Meng [27]	Hebei	2012-2013	Male		15	16.67	84	93.33	3	3.33
			Female		18	27.69	62	95.38	6	9.23
Liu [79]	Multicenter	2010-2016	Male		148	21.89	517	76.48	114	16.86
			Female		99	22.45	357	80.95	100	22.68
Zhang [72]	Yunnan	2015-2016	Male		67	67.00	98	98.00	42	42.00
			Female		63	65.63	95	98.96	39	40.63

Figure S1

Clarithromycin [By period]

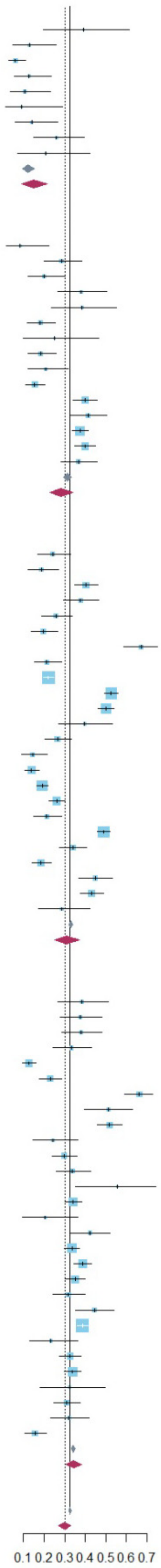

Study	Events	Total	Proportion	95%-CI	Weight (common)	Weight (random)
period = before 2005						
Wang 2019	9	23	0.39	[0.20; 0.61]	0.1%	0.9%
Gao 2010	6	47	0.13	[0.05; 0.26]	0.3%	1.1%
Zhang 2002	10	164	0.06	[0.03; 0.11]	0.9%	1.4%
Gao 2010	8	63	0.13	[0.06; 0.23]	0.3%	1.2%
Ruan 2007	5	47	0.11	[0.04; 0.23]	0.3%	1.1%
Gao 2010	2	22	0.09	[0.01; 0.29]	0.1%	0.9%
Wang 2019	7	50	0.14	[0.06; 0.27]	0.3%	1.2%
Ruan 2007	14	54	0.26	[0.15; 0.40]	0.3%	1.2%
Gao 2010	5	24	0.21	[0.07; 0.42]	0.1%	0.9%
Common effect model		494	0.12	[0.09; 0.15]	2.7%	--
Random effects model			0.15	[0.09; 0.22]	--	10.0%
Heterogeneity: $I^2 = 69\%$, $\tau^2 = 0.0107$, $p < 0.01$						
period = 2006-2010						
Gu 2007	3	36	0.08	[0.02; 0.22]	0.2%	1.1%
Ruan 2007	29	102	0.28	[0.20; 0.38]	0.6%	1.3%
Cao 2007	17	85	0.20	[0.12; 0.30]	0.5%	1.3%
Gao 2010	27	71	0.38	[0.27; 0.50]	0.4%	1.2%
Gao 2010	15	39	0.38	[0.23; 0.55]	0.2%	1.1%
Sun 2010	24	133	0.18	[0.12; 0.26]	0.7%	1.3%
Gao 2010	6	24	0.25	[0.10; 0.47]	0.1%	0.9%
Lin 2009	25	137	0.18	[0.12; 0.26]	0.7%	1.3%
Zheng 2010	16	77	0.21	[0.12; 0.32]	0.4%	1.2%
Zhou 2010	38	248	0.15	[0.11; 0.20]	1.4%	1.4%
Zhou 2014	112	280	0.40	[0.34; 0.46]	1.5%	1.4%
Qi 2021	53	128	0.41	[0.33; 0.50]	0.7%	1.3%
Song 2014	225	600	0.38	[0.34; 0.42]	3.3%	1.4%
Zhang 2015	148	371	0.40	[0.35; 0.45]	2.0%	1.4%
Tan 2011	44	120	0.37	[0.28; 0.46]	0.7%	1.3%
Common effect model		2451	0.31	[0.29; 0.33]	13.4%	--
Random effects model			0.28	[0.23; 0.34]	--	19.1%
Heterogeneity: $I^2 = 89\%$, $\tau^2 = 0.0127$, $p < 0.01$						
period = 2011-2015						
Xu 2012	29	120	0.24	[0.17; 0.33]	0.7%	1.3%
Liao 2013	21	112	0.19	[0.12; 0.27]	0.6%	1.3%
Luo 2013	121	300	0.40	[0.35; 0.46]	1.6%	1.4%
Liu 2015	49	130	0.38	[0.29; 0.47]	0.7%	1.3%
Bai 2015	37	144	0.26	[0.19; 0.34]	0.8%	1.3%
Su 2017	31	159	0.19	[0.14; 0.27]	0.9%	1.4%
Wang 2015	90	134	0.67	[0.59; 0.75]	0.7%	1.3%
Meng 2016	33	155	0.21	[0.15; 0.29]	0.8%	1.3%
Liu 2018	247	1117	0.22	[0.20; 0.25]	6.1%	1.4%
Zhang 2015	500	950	0.53	[0.49; 0.56]	5.2%	1.4%
Zhang 2015	351	700	0.50	[0.46; 0.54]	3.8%	1.4%
Song 2016	23	58	0.40	[0.27; 0.53]	0.3%	1.2%
Zhang 2015	53	200	0.26	[0.21; 0.33]	1.1%	1.4%
Hu 2016	19	132	0.14	[0.09; 0.22]	0.7%	1.3%
Hong 2016	52	374	0.14	[0.11; 0.18]	2.0%	1.4%
Liu 2019	153	804	0.19	[0.16; 0.22]	4.4%	1.4%
Pan 2020	122	467	0.26	[0.22; 0.30]	2.5%	1.4%
Zhou 2018	32	150	0.21	[0.15; 0.29]	0.8%	1.3%
Zhou 2016	464	950	0.49	[0.46; 0.52]	5.2%	1.4%
Xie 2018	70	206	0.34	[0.28; 0.41]	1.1%	1.4%
Xie 2018	53	288	0.18	[0.14; 0.23]	1.6%	1.4%
Song 2016	66	147	0.45	[0.37; 0.53]	0.8%	1.3%
Fu 2017	140	324	0.43	[0.38; 0.49]	1.8%	1.4%
Ma 2017	16	56	0.29	[0.17; 0.42]	0.3%	1.2%
Common effect model		8177	0.33	[0.32; 0.34]	44.7%	--
Random effects model			0.31	[0.25; 0.37]	--	32.7%
Heterogeneity: $I^2 = 97\%$, $\tau^2 = 0.0253$, $p < 0.01$						
period = 2016-2020						
Song 2019	25	65	0.38	[0.27; 0.51]	0.4%	1.2%
Suo 2019	36	96	0.38	[0.28; 0.48]	0.5%	1.3%
Suo 2020	38	100	0.38	[0.28; 0.48]	0.5%	1.3%
Shen 2017	35	105	0.33	[0.24; 0.43]	0.6%	1.3%
Liu 2018	50	398	0.13	[0.09; 0.16]	2.2%	1.4%
Yang 2021	56	244	0.23	[0.18; 0.29]	1.3%	1.4%
Zhang 2020	130	196	0.66	[0.59; 0.73]	1.1%	1.4%
Li 2021	38	74	0.51	[0.39; 0.63]	0.4%	1.2%
Fan 2019	140	270	0.52	[0.46; 0.58]	1.5%	1.4%
Long 2018	16	66	0.24	[0.15; 0.36]	0.4%	1.2%
Yang 2019	69	232	0.30	[0.24; 0.36]	1.3%	1.4%
Sun 2018	43	127	0.34	[0.26; 0.43]	0.7%	1.3%
Wang 2019	15	27	0.56	[0.35; 0.75]	0.1%	1.0%
Lv 2020	190	557	0.34	[0.30; 0.38]	3.0%	1.4%
Wang 2018	8	39	0.21	[0.09; 0.36]	0.2%	1.1%
Gao 2020	47	111	0.42	[0.33; 0.52]	0.6%	1.3%
Song 2020	218	650	0.34	[0.30; 0.37]	3.5%	1.4%
Cui 2021	196	506	0.39	[0.34; 0.43]	2.8%	1.4%
Chen 2019	134	382	0.35	[0.30; 0.40]	2.1%	1.4%
Yu 2019	46	145	0.32	[0.24; 0.40]	0.8%	1.3%
Tang 2020	52	117	0.44	[0.35; 0.54]	0.6%	1.3%
Jiang 2021	465	1204	0.39	[0.36; 0.41]	6.6%	1.4%
Xu 2021	13	56	0.23	[0.13; 0.36]	0.3%	1.2%
Zhang 2021	103	315	0.33	[0.28; 0.38]	1.7%	1.4%
Jiang 2020	187	553	0.34	[0.30; 0.38]	3.0%	1.4%
Luo 2020	12	37	0.32	[0.18; 0.50]	0.2%	1.1%
Luo 2020	64	207	0.31	[0.25; 0.38]	1.1%	1.4%
Xu 2020	32	100	0.32	[0.23; 0.42]	0.5%	1.3%
He 2021	31	200	0.16	[0.11; 0.21]	1.1%	1.4%
Common effect model		7179	0.34	[0.33; 0.35]	39.2%	--
Random effects model			0.34	[0.30; 0.39]	--	38.2%
Heterogeneity: $I^2 = 92\%$, $\tau^2 = 0.0114$, $p < 0.01$						
**Common effect model**		**18301**	**0.33**	**[0.32; 0.33]**	**100.0%**	**--**
**Random effects model**			**0.30**	**[0.27; 0.33]**	**--**	**100.0%**
Heterogeneity: $I^2 = 95\%$, $\tau^2 = 0.0185$, $p < 0.01$						

Figure S2

Clarithromycin [By region]

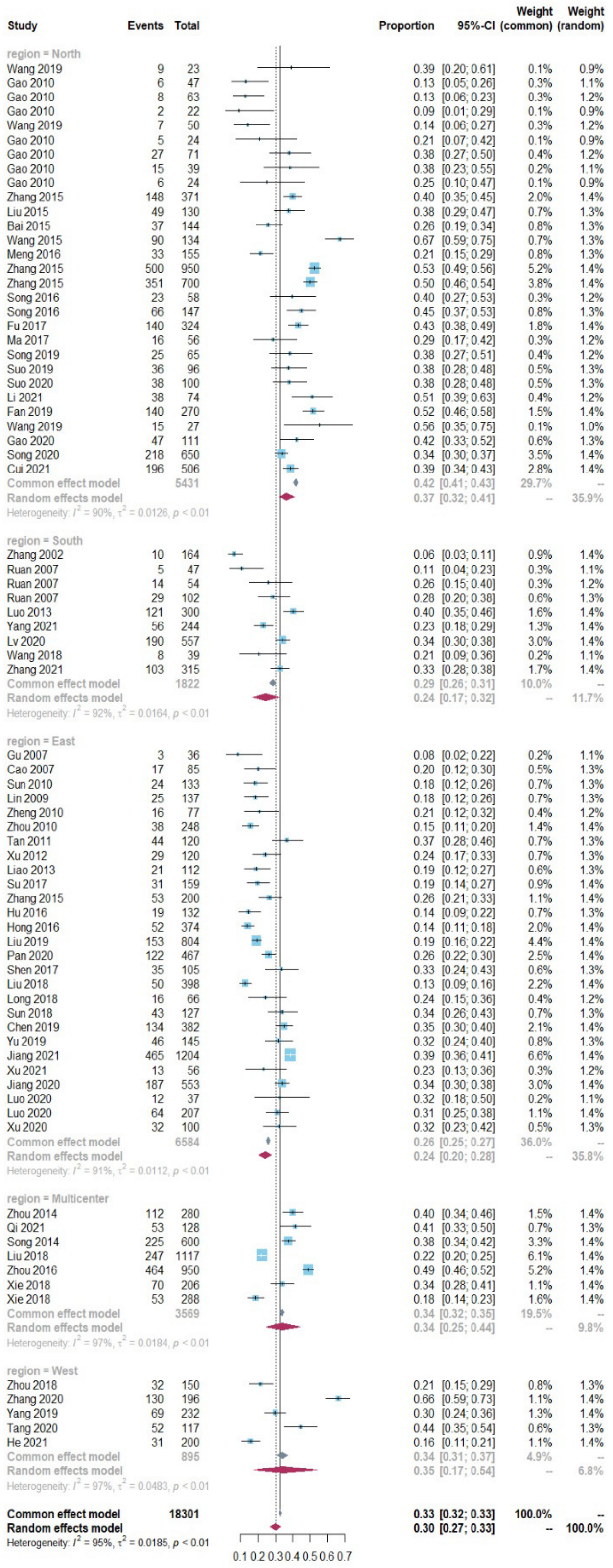

Figure S3

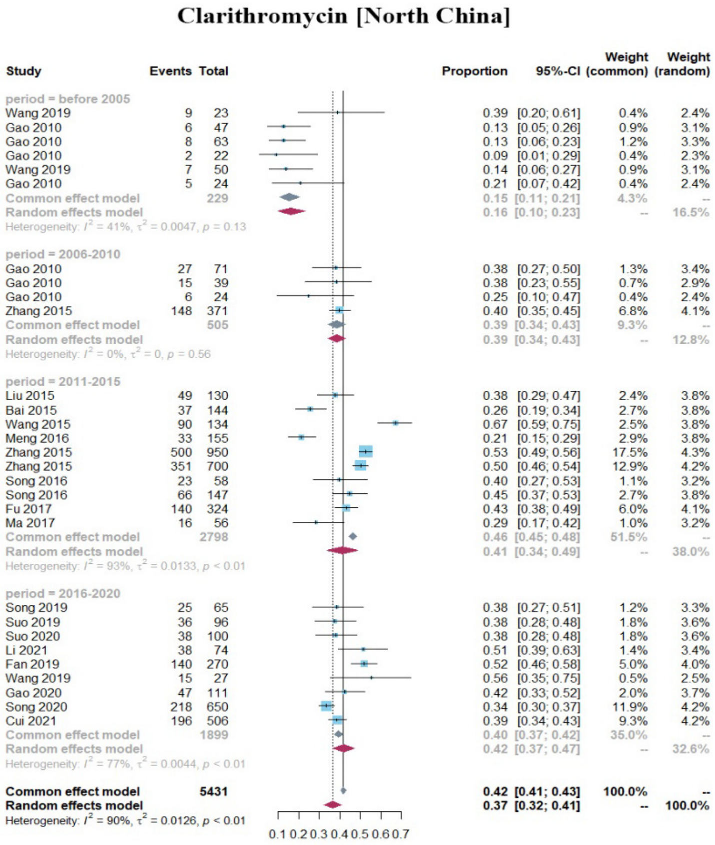
Clarithromycin [North China]
Study Events Total Proportion 95%-CI Weight (common) Weight (random)
period = before 2005
Wang 2019 9 23 0.39 [0.20; 0.61] 0.4% 2.4%
Gao 2010 6 47 0.13 [0.05; 0.26] 0.9% 3.1%
Gao 2010 8 63 0.13 [0.06; 0.23] 1.2% 3.3%
Gao 2010 2 22 0.09 [0.01; 0.29] 0.4% 2.3%
Wang 2019 7 50 0.14 [0.06; 0.27] 0.9% 3.1%
Gao 2010 5 24 0.21 [0.07; 0.42] 0.4% 2.4%
Common effect model 229 0.15 [0.11; 0.21] 4.3% --
Random effects model 0.16 [0.10; 0.23] -- 16.5%
Heterogeneity: I^2 = 41%, τ^2 = 0.0047, p = 0.13
period = 2006-2010
Gao 2010 27 71 0.38 [0.27; 0.50] 1.3% 3.4%
Gao 2010 15 39 0.38 [0.23; 0.55] 0.7% 2.9%
Gao 2010 6 24 0.25 [0.10; 0.47] 0.4% 2.4%
Zhang 2015 148 371 0.40 [0.35; 0.45] 6.8% 4.1%
Common effect model 505 0.39 [0.34; 0.43] 9.3% --
Random effects model 0.39 [0.34; 0.43] -- 12.8%
Heterogeneity: I^2 = 0%, τ^2 = 0, p = 0.56
period = 2011-2015
Liu 2015 49 130 0.38 [0.29; 0.47] 2.4% 3.8%
Bai 2015 37 144 0.26 [0.19; 0.34] 2.7% 3.8%
Wang 2015 90 134 0.67 [0.59; 0.75] 2.5% 3.8%
Meng 2016 33 155 0.21 [0.15; 0.29] 2.9% 3.8%
Zhang 2015 500 950 0.53 [0.49; 0.56] 17.5% 4.3%
Zhang 2015 351 700 0.50 [0.46; 0.54] 12.9% 4.2%
Song 2016 23 58 0.40 [0.27; 0.53] 1.1% 3.2%
Song 2016 66 147 0.45 [0.37; 0.53] 2.7% 3.8%
Fu 2017 140 324 0.43 [0.38; 0.49] 6.0% 4.1%
Ma 2017 16 56 0.29 [0.17; 0.42] 1.0% 3.2%
Common effect model 2798 0.46 [0.45; 0.48] 51.5% --
Random effects model 0.41 [0.34; 0.49] -- 38.0%
Heterogeneity: I^2 = 93%, τ^2 = 0.0133, p < 0.01
period = 2016-2020
Song 2019 25 65 0.38 [0.27; 0.51] 1.2% 3.3%
Suo 2019 36 96 0.38 [0.28; 0.48] 1.8% 3.6%
Suo 2020 38 100 0.38 [0.28; 0.48] 1.8% 3.6%
Li 2021 38 74 0.51 [0.39; 0.63] 1.4% 3.4%
Fan 2019 140 270 0.52 [0.46; 0.58] 5.0% 4.0%
Wang 2019 15 27 0.56 [0.35; 0.75] 0.5% 2.5%
Gao 2020 47 111 0.42 [0.33; 0.52] 2.0% 3.7%
Song 2020 218 650 0.34 [0.30; 0.37] 11.9% 4.2%
Cui 2021 196 506 0.39 [0.34; 0.43] 9.3% 4.2%
Common effect model 1899 0.40 [0.37; 0.42] 35.0% --
Random effects model 0.42 [0.37; 0.47] -- 32.6%
Heterogeneity: I^2 = 77%, τ^2 = 0.0044, p < 0.01
Common effect model 5431 0.42 [0.41; 0.43] 100.0% --
Random effects model 0.37 [0.32; 0.41] -- 100.0%
Heterogeneity: I^2 = 90%, τ^2 = 0.0126, p < 0.01
0.1 0.2 0.3 0.4 0.5 0.6 0.7

Figure S4

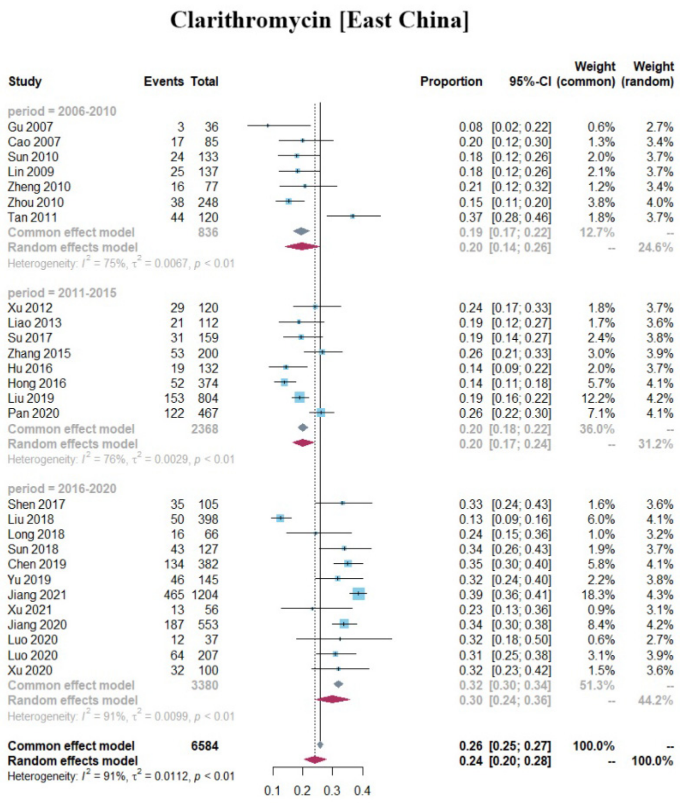
Clarithromycin [East China]
Study Events Total Proportion 95%-CI Weight (common) Weight (random)
period = 2006-2010
Gu 2007 3 36 0.08 [0.02; 0.22] 0.6% 2.7%
Cao 2007 17 85 0.20 [0.12; 0.30] 1.3% 3.4%
Sun 2010 24 133 0.18 [0.12; 0.26] 2.0% 3.7%
Lin 2009 25 137 0.18 [0.12; 0.26] 2.1% 3.7%
Zheng 2010 16 77 0.21 [0.12; 0.32] 1.2% 3.4%
Zhou 2010 38 248 0.15 [0.11; 0.20] 3.8% 4.0%
Tan 2011 44 120 0.37 [0.28; 0.46] 1.8% 3.7%
Common effect model 836 0.19 [0.17; 0.22] 12.7% --
Random effects model 0.20 [0.14; 0.26] -- 24.6%
Heterogeneity: $I^2 = 75\%$, $\tau^2 = 0.0067$, $p < 0.01$
period = 2011-2015
Xu 2012 29 120 0.24 [0.17; 0.33] 1.8% 3.7%
Liao 2013 21 112 0.19 [0.12; 0.27] 1.7% 3.6%
Su 2017 31 159 0.19 [0.14; 0.27] 2.4% 3.8%
Zhang 2015 53 200 0.26 [0.21; 0.33] 3.0% 3.9%
Hu 2016 19 132 0.14 [0.09; 0.22] 2.0% 3.7%
Hong 2016 52 374 0.14 [0.11; 0.18] 5.7% 4.1%
Liu 2019 153 804 0.19 [0.16; 0.22] 12.2% 4.2%
Pan 2020 122 467 0.26 [0.22; 0.30] 7.1% 4.1%
Common effect model 2368 0.20 [0.18; 0.22] 36.0% --
Random effects model 0.20 [0.17; 0.24] -- 31.2%
Heterogeneity: $I^2 = 76\%$, $\tau^2 = 0.0029$, $p < 0.01$
period = 2016-2020
Shen 2017 35 105 0.33 [0.24; 0.43] 1.6% 3.6%
Liu 2018 50 398 0.13 [0.09; 0.16] 6.0% 4.1%
Long 2018 16 66 0.24 [0.15; 0.36] 1.0% 3.2%
Sun 2018 43 127 0.34 [0.26; 0.43] 1.9% 3.7%
Chen 2019 134 382 0.35 [0.30; 0.40] 5.8% 4.1%
Yu 2019 46 145 0.32 [0.24; 0.40] 2.2% 3.8%
Jiang 2021 465 1204 0.39 [0.36; 0.41] 18.3% 4.3%
Xu 2021 13 56 0.23 [0.13; 0.36] 0.9% 3.1%
Jiang 2020 187 553 0.34 [0.30; 0.38] 8.4% 4.2%
Luo 2020 12 37 0.32 [0.18; 0.50] 0.6% 2.7%
Luo 2020 64 207 0.31 [0.25; 0.38] 3.1% 3.9%
Xu 2020 32 100 0.32 [0.23; 0.42] 1.5% 3.6%
Common effect model 3380 0.32 [0.30; 0.34] 51.3% --
Random effects model 0.30 [0.24; 0.36] -- 44.2%
Heterogeneity: $I^2 = 91\%$, $\tau^2 = 0.0099$, $p < 0.01$
Common effect model 6584 0.26 [0.25; 0.27] 100.0% --
Random effects model 0.24 [0.20; 0.28] -- 100.0%
Heterogeneity: $I^2 = 91\%$, $\tau^2 = 0.0112$, $p < 0.01$
0.1 0.2 0.3 0.4

Figure S5

Metronidazole [By period]

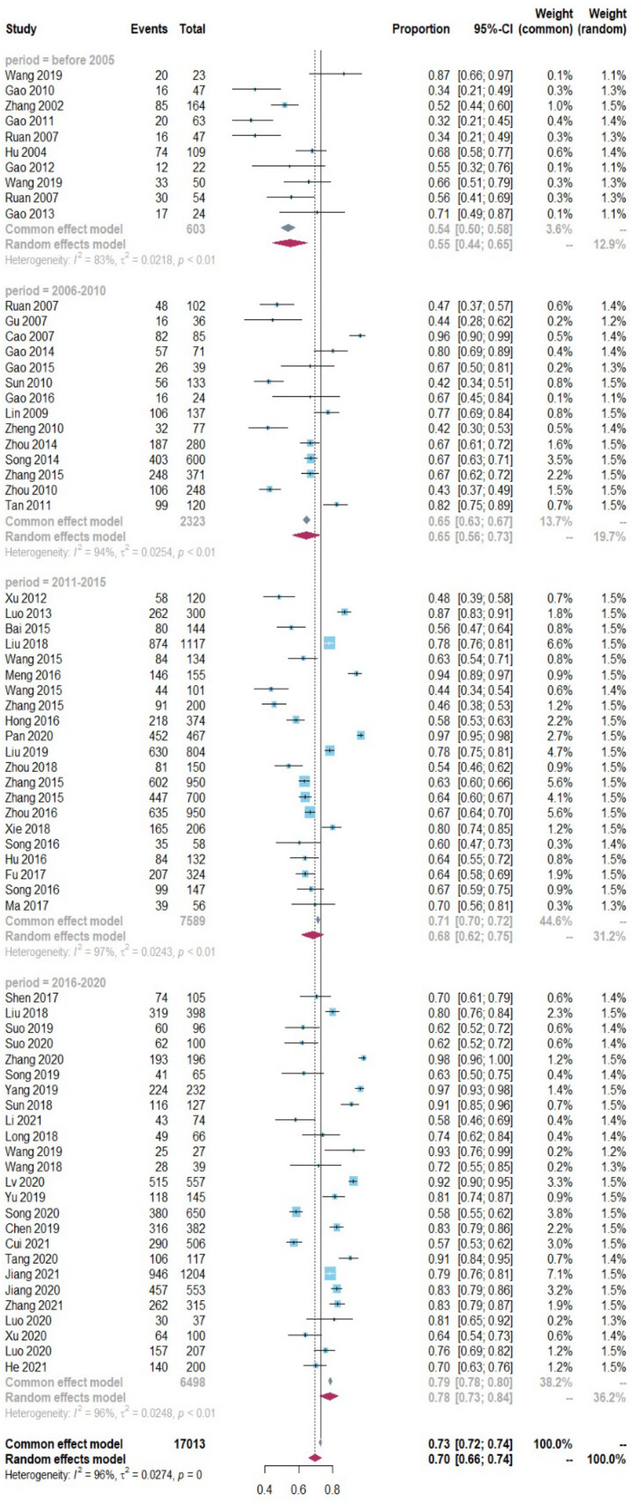

Figure S6

Metronidazole [By region]

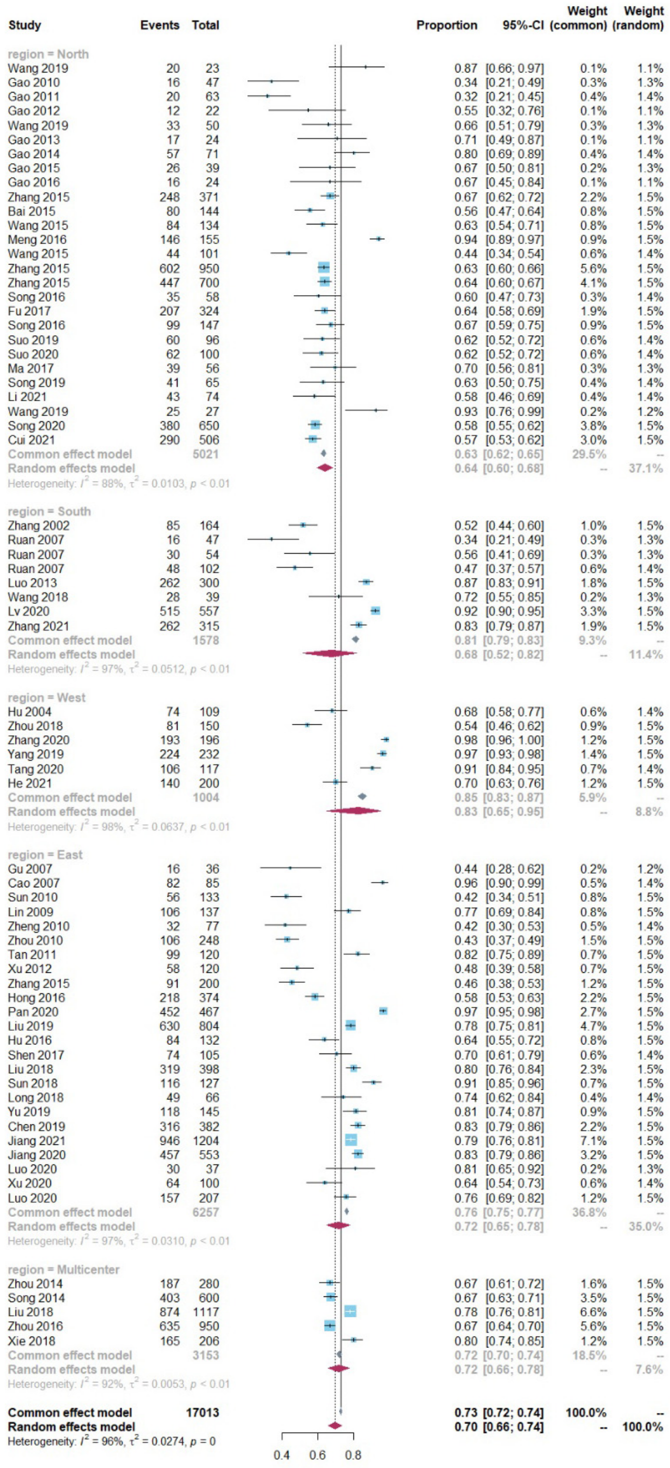

Figure S7

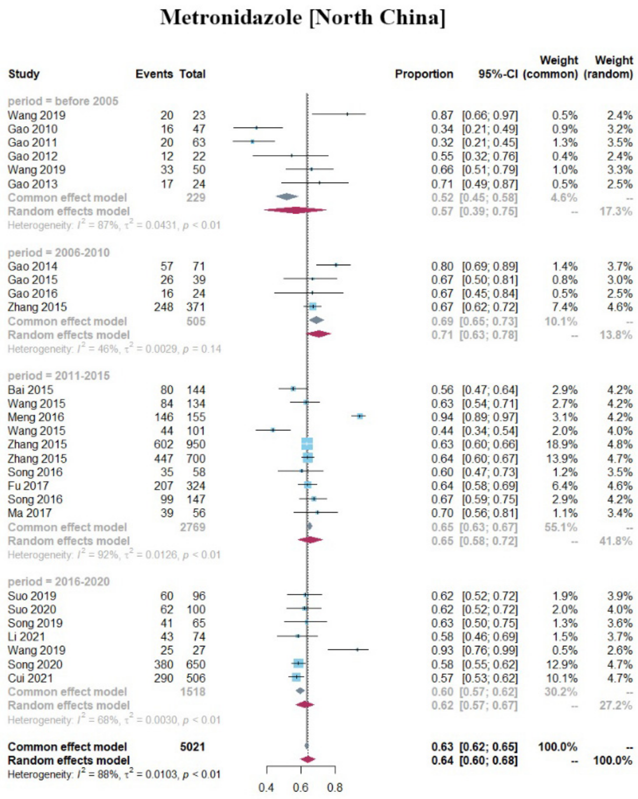
Metronidazole [North China]
Study Events Total Proportion 95%-CI Weight (common) Weight (random)
period = before 2005
Wang 2019 20 23 0.87 [0.66; 0.97] 0.5% 2.4%
Gao 2010 16 47 0.34 [0.21; 0.49] 0.9% 3.2%
Gao 2011 20 63 0.32 [0.21; 0.45] 1.3% 3.5%
Gao 2012 12 22 0.55 [0.32; 0.76] 0.4% 2.4%
Wang 2019 33 50 0.66 [0.51; 0.79] 1.0% 3.3%
Gao 2013 17 24 0.71 [0.49; 0.87] 0.5% 2.5%
Common effect model 229 0.52 [0.45; 0.58] 4.6% --
Random effects model 0.57 [0.39; 0.75] -- 17.3%
Heterogeneity: I^2 = 87%, τ^2 = 0.0431, p < 0.01
period = 2006-2010
Gao 2014 57 71 0.80 [0.69; 0.89] 1.4% 3.7%
Gao 2015 26 39 0.67 [0.50; 0.81] 0.8% 3.0%
Gao 2016 16 24 0.67 [0.45; 0.84] 0.5% 2.5%
Zhang 2015 248 371 0.67 [0.62; 0.72] 7.4% 4.6%
Common effect model 505 0.69 [0.65; 0.73] 10.1% --
Random effects model 0.71 [0.63; 0.78] -- 13.8%
Heterogeneity: I^2 = 46%, τ^2 = 0.0029, p = 0.14
period = 2011-2015
Bai 2015 80 144 0.56 [0.47; 0.64] 2.9% 4.2%
Wang 2015 84 134 0.63 [0.54; 0.71] 2.7% 4.2%
Meng 2016 146 155 0.94 [0.89; 0.97] 3.1% 4.2%
Wang 2015 44 101 0.44 [0.34; 0.54] 2.0% 4.0%
Zhang 2015 602 950 0.63 [0.60; 0.66] 18.9% 4.8%
Zhang 2015 447 700 0.64 [0.60; 0.67] 13.9% 4.7%
Song 2016 35 58 0.60 [0.47; 0.73] 1.2% 3.5%
Fu 2017 207 324 0.64 [0.58; 0.69] 6.4% 4.6%
Song 2016 99 147 0.67 [0.59; 0.75] 2.9% 4.2%
Ma 2017 39 56 0.70 [0.56; 0.81] 1.1% 3.4%
Common effect model 2769 0.65 [0.63; 0.67] 55.1% --
Random effects model 0.65 [0.58; 0.72] -- 41.8%
Heterogeneity: I^2 = 92%, τ^2 = 0.0126, p < 0.01
period = 2016-2020
Suo 2019 60 96 0.62 [0.52; 0.72] 1.9% 3.9%
Suo 2020 62 100 0.62 [0.52; 0.72] 2.0% 4.0%
Song 2019 41 65 0.63 [0.50; 0.75] 1.3% 3.6%
Li 2021 43 74 0.58 [0.46; 0.69] 1.5% 3.7%
Wang 2019 25 27 0.93 [0.76; 0.99] 0.5% 2.6%
Song 2020 380 650 0.58 [0.55; 0.62] 12.9% 4.7%
Cui 2021 290 506 0.57 [0.53; 0.62] 10.1% 4.7%
Common effect model 1518 0.60 [0.57; 0.62] 30.2% --
Random effects model 0.62 [0.57; 0.67] -- 27.2%
Heterogeneity: I^2 = 68%, τ^2 = 0.0030, p < 0.01
Common effect model 5021 0.63 [0.62; 0.65] 100.0% --
Random effects model 0.64 [0.60; 0.68] -- 100.0%
Heterogeneity: I^2 = 88%, τ^2 = 0.0103, p < 0.01
0.4 0.6 0.8

Figure S8

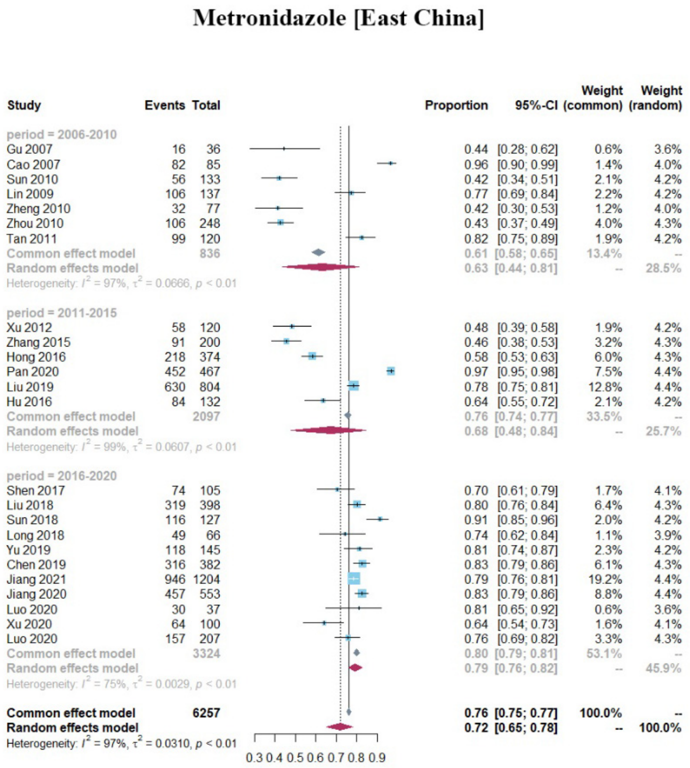
Metronidazole [East China]
Study | Events | Total | Proportion | 95%-CI | Weight (common) | Weight (random)
period = 2006-2010
Gu 2007 16 36 0.44 [0.28; 0.62] 0.6% 3.6%
Cao 2007 82 85 0.96 [0.90; 0.99] 1.4% 4.0%
Sun 2010 56 133 0.42 [0.34; 0.51] 2.1% 4.2%
Lin 2009 106 137 0.77 [0.69; 0.84] 2.2% 4.2%
Zheng 2010 32 77 0.42 [0.30; 0.53] 1.2% 4.0%
Zhou 2010 106 248 0.43 [0.37; 0.49] 4.0% 4.3%
Tan 2011 99 120 0.82 [0.75; 0.89] 1.9% 4.2%
Common effect model 836 0.61 [0.58; 0.65] 13.4% --
Random effects model 0.63 [0.44; 0.81] -- 28.5%
Heterogeneity: I^2 = 97%, τ^2 = 0.0666, p < 0.01
period = 2011-2015
Xu 2012 58 120 0.48 [0.39; 0.58] 1.9% 4.2%
Zhang 2015 91 200 0.46 [0.38; 0.53] 3.2% 4.3%
Hong 2016 218 374 0.58 [0.53; 0.63] 6.0% 4.3%
Pan 2020 452 467 0.97 [0.95; 0.98] 7.5% 4.4%
Liu 2019 630 804 0.78 [0.75; 0.81] 12.8% 4.4%
Hu 2016 84 132 0.64 [0.55; 0.72] 2.1% 4.2%
Common effect model 2097 0.76 [0.74; 0.77] 33.5% --
Random effects model 0.68 [0.48; 0.84] -- 25.7%
Heterogeneity: I^2 = 99%, τ^2 = 0.0607, p < 0.01
period = 2016-2020
Shen 2017 74 105 0.70 [0.61; 0.79] 1.7% 4.1%
Liu 2018 319 398 0.80 [0.76; 0.84] 6.4% 4.3%
Sun 2018 116 127 0.91 [0.85; 0.96] 2.0% 4.2%
Long 2018 49 66 0.74 [0.62; 0.84] 1.1% 3.9%
Yu 2019 118 145 0.81 [0.74; 0.87] 2.3% 4.2%
Chen 2019 316 382 0.83 [0.79; 0.86] 6.1% 4.3%
Jiang 2021 946 1204 0.79 [0.76; 0.81] 19.2% 4.4%
Jiang 2020 457 553 0.83 [0.79; 0.86] 8.8% 4.4%
Luo 2020 30 37 0.81 [0.65; 0.92] 0.6% 3.6%
Xu 2020 64 100 0.64 [0.54; 0.73] 1.6% 4.1%
Luo 2020 157 207 0.76 [0.69; 0.82] 3.3% 4.3%
Common effect model 3324 0.80 [0.79; 0.81] 53.1% --
Random effects model 0.79 [0.76; 0.82] -- 45.9%
Heterogeneity: I^2 = 75%, τ^2 = 0.0029, p < 0.01
Common effect model 6257 0.76 [0.75; 0.77] 100.0% --
Random effects model 0.72 [0.65; 0.78] -- 100.0%
Heterogeneity: I^2 = 97%, τ^2 = 0.0310, p < 0.01
0.3 0.4 0.5 0.6 0.7 0.8 0.9

Figure S9

Levofloxacin [By period]

Study	Events	Total	Proportion	95%-CI	Weight (common)	Weight (random)
period = 2006-2010						
Gao 2010	10	40	0.25	[0.13; 0.41]	0.3%	1.7%
Gao 2010	18	39	0.46	[0.30; 0.63]	0.3%	1.7%
Gao 2010	10	24	0.42	[0.22; 0.63]	0.2%	1.5%
Zhang 2015	128	371	0.35	[0.30; 0.40]	2.6%	2.0%
Zhou 2014	4	100	0.04	[0.01; 0.10]	0.7%	1.9%
Lin 2009	41	137	0.30	[0.22; 0.38]	1.0%	2.0%
Tan 2011	50	120	0.42	[0.33; 0.51]	0.8%	1.9%
Song 2014	201	600	0.34	[0.30; 0.37]	4.2%	2.1%
Common effect model		1431	0.31	[0.29; 0.34]	10.1%	--
Random effects model			0.30	[0.22; 0.39]	--	14.7%
Heterogeneity: $I^2 = 90\%$, $\tau^2 = 0.0156$, $p < 0.01$						
period = 2011-2015						
Wang 2015	76	134	0.57	[0.48; 0.65]	0.9%	1.9%
Meng 2016	9	155	0.06	[0.03; 0.11]	1.1%	2.0%
Liao 2013	34	112	0.30	[0.22; 0.40]	0.8%	1.9%
Luo 2013	20	300	0.07	[0.04; 0.10]	2.1%	2.0%
Zhou 2014	12	100	0.12	[0.06; 0.20]	0.7%	1.9%
Su 2017	49	159	0.31	[0.24; 0.39]	1.1%	2.0%
Zhang 2015	521	950	0.55	[0.52; 0.58]	6.7%	2.1%
Zhang 2015	381	700	0.54	[0.51; 0.58]	4.9%	2.1%
Liu	214	1117	0.19	[0.17; 0.22]	7.8%	2.1%
Hong 2016	47	374	0.13	[0.09; 0.16]	2.6%	2.0%
Pan 2020	134	467	0.29	[0.25; 0.33]	3.3%	2.0%
Zhou 2018	6	150	0.04	[0.01; 0.09]	1.1%	2.0%
Liu 2019	187	804	0.23	[0.20; 0.26]	5.6%	2.1%
Hu 2016	40	132	0.30	[0.23; 0.39]	0.9%	1.9%
Song 2016	21	58	0.36	[0.24; 0.50]	0.4%	1.8%
Fu 2017	147	324	0.45	[0.40; 0.51]	2.3%	2.0%
Ma 2017	0	56	0.00	[0.00; 0.06]	0.4%	1.8%
Common effect model		6092	0.29	[0.28; 0.31]	42.8%	--
Random effects model			0.24	[0.16; 0.33]	--	33.6%
Heterogeneity: $I^2 = 98\%$, $\tau^2 = 0.0453$, $p < 0.01$						
period = 2016-2020						
Suo 2019	36	96	0.38	[0.28; 0.48]	0.7%	1.9%
Shen 2017	34	105	0.32	[0.24; 0.42]	0.7%	1.9%
Liu 2018	154	398	0.39	[0.34; 0.44]	2.8%	2.0%
Yang 2021	13	244	0.05	[0.03; 0.09]	1.7%	2.0%
Zhang 2020	81	196	0.41	[0.34; 0.49]	1.4%	2.0%
Suo 2020	39	100	0.39	[0.29; 0.49]	0.7%	1.9%
Song 2019	26	65	0.40	[0.28; 0.53]	0.5%	1.8%
Li 2021	21	74	0.28	[0.19; 0.40]	0.5%	1.8%
Wang 2019	22	27	0.81	[0.62; 0.94]	0.2%	1.5%
Sun 2018	57	127	0.45	[0.36; 0.54]	0.9%	1.9%
Gao 2020	46	111	0.41	[0.32; 0.51]	0.8%	1.9%
Yang 2019	88	232	0.38	[0.32; 0.45]	1.6%	2.0%
Wang 2018	2	39	0.05	[0.01; 0.17]	0.3%	1.7%
Song 2020	219	650	0.34	[0.30; 0.37]	4.6%	2.1%
Lv 2020	236	557	0.42	[0.38; 0.47]	3.9%	2.1%
Yu 2019	59	145	0.41	[0.33; 0.49]	1.0%	2.0%
Cui 2021	157	506	0.31	[0.27; 0.35]	3.6%	2.1%
Chen 2019	179	382	0.47	[0.42; 0.52]	2.7%	2.0%
Tang 2020	33	117	0.28	[0.20; 0.37]	0.8%	1.9%
Jiang 2021	330	1204	0.27	[0.25; 0.30]	8.4%	2.1%
Jiang 2020	103	553	0.19	[0.15; 0.22]	3.9%	2.1%
He 2021	85	262	0.32	[0.27; 0.38]	1.8%	2.0%
Luo 2020	17	37	0.46	[0.29; 0.63]	0.3%	1.7%
Luo 2020	87	207	0.42	[0.35; 0.49]	1.5%	2.0%
He 2021	77	200	0.38	[0.32; 0.46]	1.4%	2.0%
Common effect model		6634	0.33	[0.31; 0.34]	46.6%	--
Random effects model			0.35	[0.30; 0.40]	--	48.4%
Heterogeneity: $I^2 = 93\%$, $\tau^2 = 0.0139$, $p < 0.01$						
period = before 2005						
Wang 2019	11	23	0.48	[0.27; 0.69]	0.2%	1.5%
Wang 2019	23	50	0.46	[0.32; 0.61]	0.4%	1.8%
Common effect model		73	0.47	[0.35; 0.58]	0.5%	--
Random effects model			0.47	[0.35; 0.58]	--	3.2%
Heterogeneity: $I^2 = 0\%$, $\tau^2 = 0$, $p = 0.88$						
**Common effect model**		**14230**	**0.31**	**[0.30; 0.32]**	**100.0%**	**--**
**Random effects model**			**0.31**	**[0.27; 0.35]**	**--**	**100.0%**
Heterogeneity: $I^2 = 97\%$, $\tau^2 = 0.0258$, $p < 0.01$						

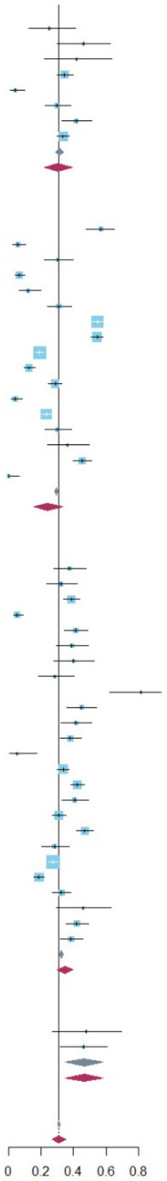

Figure S10

Levofloxacin [By region]

Study	Events	Total	Proportion	95%-CI	Weight (common)	Weight (random)
region = North						
Gao 2010	10	40	0.25	[0.13; 0.41]	0.3%	1.7%
Gao 2010	18	39	0.46	[0.30; 0.63]	0.3%	1.7%
Gao 2010	10	24	0.42	[0.22; 0.63]	0.2%	1.5%
Zhang 2015	128	371	0.35	[0.30; 0.40]	2.6%	2.0%
Wang 2015	76	134	0.57	[0.48; 0.65]	0.9%	1.9%
Meng 2016	9	155	0.06	[0.03; 0.11]	1.1%	2.0%
Zhang 2015	521	950	0.55	[0.52; 0.58]	6.7%	2.1%
Zhang 2015	381	700	0.54	[0.51; 0.58]	4.9%	2.1%
Song 2016	21	58	0.36	[0.24; 0.50]	0.4%	1.8%
Fu 2017	147	324	0.45	[0.40; 0.51]	2.3%	2.0%
Ma 2017	0	56	0.00	[0.00; 0.06]	0.4%	1.8%
Suo 2019	36	96	0.38	[0.28; 0.48]	0.7%	1.9%
Suo 2020	39	100	0.39	[0.29; 0.49]	0.7%	1.9%
Song 2019	26	65	0.40	[0.28; 0.53]	0.5%	1.8%
Li 2021	21	74	0.28	[0.19; 0.40]	0.5%	1.8%
Wang 2019	22	27	0.81	[0.62; 0.94]	0.2%	1.5%
Gao 2020	46	111	0.41	[0.32; 0.51]	0.8%	1.9%
Song 2020	219	650	0.34	[0.30; 0.37]	4.6%	2.1%
Cui 2021	157	506	0.31	[0.27; 0.35]	3.6%	2.1%
Wang 2019	11	23	0.48	[0.27; 0.69]	0.2%	1.5%
Wang 2019	23	50	0.46	[0.32; 0.61]	0.4%	1.8%
Common effect model		4553	0.41	[0.40; 0.43]	32.0%	--
Random effects model			0.38	[0.31; 0.45]	--	38.8%
Heterogeneity: $I^2 = 95\%$, $\tau^2 = 0.0252$, $p < 0.01$						
region = West						
Zhou 2014	4	100	0.04	[0.01; 0.10]	0.7%	1.9%
Zhou 2014	12	100	0.12	[0.06; 0.20]	0.7%	1.9%
Zhou 2018	6	150	0.04	[0.01; 0.09]	1.1%	2.0%
Zhang 2020	81	196	0.41	[0.34; 0.49]	1.4%	2.0%
Yang 2019	88	232	0.38	[0.32; 0.45]	1.6%	2.0%
Tang 2020	33	117	0.28	[0.20; 0.37]	0.8%	1.9%
He 2021	77	200	0.38	[0.32; 0.46]	1.4%	2.0%
Common effect model		1095	0.25	[0.23; 0.28]	7.7%	--
Random effects model			0.21	[0.10; 0.36]	--	13.7%
Heterogeneity: $I^2 = 96\%$, $\tau^2 = 0.0442$, $p < 0.01$						
region = East						
Lin 2009	41	137	0.30	[0.22; 0.38]	1.0%	2.0%
Tan 2011	50	120	0.42	[0.33; 0.51]	0.8%	1.9%
Liao 2013	34	112	0.30	[0.22; 0.40]	0.8%	1.9%
Su 2017	49	159	0.31	[0.24; 0.39]	1.1%	2.0%
Hong 2016	47	374	0.13	[0.09; 0.16]	2.6%	2.0%
Pan 2020	134	467	0.29	[0.25; 0.33]	3.3%	2.0%
Liu 2019	187	804	0.23	[0.20; 0.26]	5.6%	2.1%
Hu 2016	40	132	0.30	[0.23; 0.39]	0.9%	1.9%
Shen 2017	34	105	0.32	[0.24; 0.42]	0.7%	1.9%
Liu 2018	154	398	0.39	[0.34; 0.44]	2.8%	2.0%
Sun 2018	57	127	0.45	[0.36; 0.54]	0.9%	1.9%
Yu 2019	59	145	0.41	[0.33; 0.49]	1.0%	2.0%
Chen 2019	179	382	0.47	[0.42; 0.52]	2.7%	2.0%
Jiang 2021	330	1204	0.27	[0.25; 0.30]	8.4%	2.1%
Jiang 2020	103	553	0.19	[0.15; 0.22]	3.9%	2.1%
Luo 2020	17	37	0.46	[0.29; 0.63]	0.3%	1.7%
Luo 2020	87	207	0.42	[0.35; 0.49]	1.5%	2.0%
Common effect model		5463	0.29	[0.28; 0.30]	38.4%	--
Random effects model			0.32	[0.27; 0.37]	--	33.6%
Heterogeneity: $I^2 = 93\%$, $\tau^2 = 0.0111$, $p < 0.01$						
region = Multicenter						
Song 2014	201	600	0.34	[0.30; 0.37]	4.2%	2.1%
Liu	214	1117	0.19	[0.17; 0.22]	7.8%	2.1%
Common effect model		1717	0.24	[0.22; 0.26]	12.1%	--
Random effects model			0.26	[0.13; 0.41]	--	4.1%
Heterogeneity: $I^2 = 98\%$, $\tau^2 = 0.0132$, $p < 0.01$						
region = South						
Luo 2013	20	300	0.07	[0.04; 0.10]	2.1%	2.0%
Yang 2021	13	244	0.05	[0.03; 0.09]	1.7%	2.0%
Wang 2018	2	39	0.05	[0.01; 0.17]	0.3%	1.7%
Lv 2020	236	557	0.42	[0.38; 0.47]	3.9%	2.1%
He 2021	85	262	0.32	[0.27; 0.38]	1.8%	2.0%
Common effect model		1402	0.23	[0.21; 0.25]	9.9%	--
Random effects model			0.16	[0.03; 0.35]	--	9.8%
Heterogeneity: $I^2 = 98\%$, $\tau^2 = 0.0608$, $p < 0.01$						
**Common effect model**		**14230**	**0.31**	**[0.30; 0.32]**	**100.0%**	**--**
**Random effects model**			**0.31**	**[0.27; 0.35]**	**--**	**100.0%**
Heterogeneity: $I^2 = 97\%$, $\tau^2 = 0.0258$, $p < 0.01$						

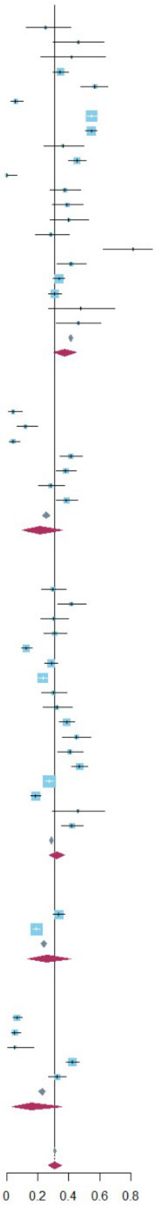

Figure S11

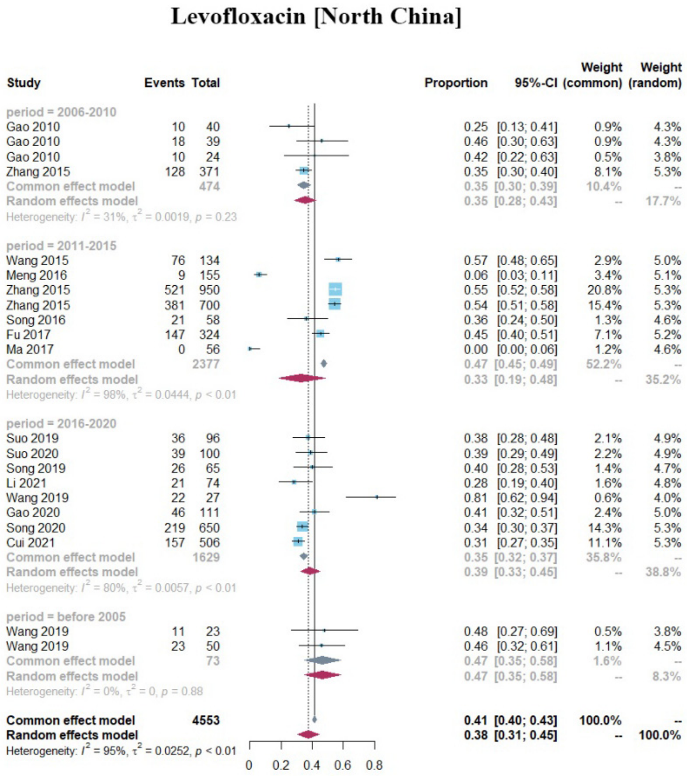
Levofloxacin [North China]
Study Events Total Proportion 95%-CI Weight (common) Weight (random)
period = 2006-2010
Gao 2010 10 40 0.25 [0.13; 0.41] 0.9% 4.3%
Gao 2010 18 39 0.46 [0.30; 0.63] 0.9% 4.3%
Gao 2010 10 24 0.42 [0.22; 0.63] 0.5% 3.8%
Zhang 2015 128 371 0.35 [0.30; 0.40] 8.1% 5.3%
Common effect model 474 0.35 [0.30; 0.39] 10.4% --
Random effects model 0.35 [0.28; 0.43] -- 17.7%
Heterogeneity: I^2 = 31%, τ^2 = 0.0019, p = 0.23
period = 2011-2015
Wang 2015 76 134 0.57 [0.48; 0.65] 2.9% 5.0%
Meng 2016 9 155 0.06 [0.03; 0.11] 3.4% 5.1%
Zhang 2015 521 950 0.55 [0.52; 0.58] 20.8% 5.3%
Zhang 2015 381 700 0.54 [0.51; 0.58] 15.4% 5.3%
Song 2016 21 58 0.36 [0.24; 0.50] 1.3% 4.6%
Fu 2017 147 324 0.45 [0.40; 0.51] 7.1% 5.2%
Ma 2017 0 56 0.00 [0.00; 0.06] 1.2% 4.6%
Common effect model 2377 0.47 [0.45; 0.49] 52.2% --
Random effects model 0.33 [0.19; 0.48] -- 35.2%
Heterogeneity: I^2 = 98%, τ^2 = 0.0444, p < 0.01
period = 2016-2020
Suo 2019 36 96 0.38 [0.28; 0.48] 2.1% 4.9%
Suo 2020 39 100 0.39 [0.29; 0.49] 2.2% 4.9%
Song 2019 26 65 0.40 [0.28; 0.53] 1.4% 4.7%
Li 2021 21 74 0.28 [0.19; 0.40] 1.6% 4.8%
Wang 2019 22 27 0.81 [0.62; 0.94] 0.6% 4.0%
Gao 2020 46 111 0.41 [0.32; 0.51] 2.4% 5.0%
Song 2020 219 650 0.34 [0.30; 0.37] 14.3% 5.3%
Cui 2021 157 506 0.31 [0.27; 0.35] 11.1% 5.3%
Common effect model 1629 0.35 [0.32; 0.37] 35.8% --
Random effects model 0.39 [0.33; 0.45] -- 38.8%
Heterogeneity: I^2 = 80%, τ^2 = 0.0057, p < 0.01
period = before 2005
Wang 2019 11 23 0.48 [0.27; 0.69] 0.5% 3.8%
Wang 2019 23 50 0.46 [0.32; 0.61] 1.1% 4.5%
Common effect model 73 0.47 [0.35; 0.58] 1.6% --
Random effects model 0.47 [0.35; 0.58] -- 8.3%
Heterogeneity: I^2 = 0%, τ^2 = 0, p = 0.88
Common effect model 4553 0.41 [0.40; 0.43] 100.0% --
Random effects model 0.38 [0.31; 0.45] -- 100.0%
Heterogeneity: I^2 = 95%, τ^2 = 0.0252, p < 0.01
0 0.2 0.4 0.6 0.8

Figure S12

Levofloxacin [East China]

Study	Events	Total	Proportion	95%-CI	Weight (common)	Weight (random)
period = 2006-2010						
Lin 2009	41	137	0.30	[0.22; 0.38]	2.5%	5.7%
Tan 2011	50	120	0.42	[0.33; 0.51]	2.2%	5.6%
Common effect model		257	0.35	[0.30; 0.41]	4.7%	--
Random effects model			0.36	[0.25; 0.47]	--	11.3%
Heterogeneity: $I^2 = 74\%$, $\tau^2 = 0.0055$, $p = 0.05$						
period = 2011-2015						
Liao 2013	34	112	0.30	[0.22; 0.40]	2.1%	5.5%
Su 2017	49	159	0.31	[0.24; 0.39]	2.9%	5.8%
Hong 2016	47	374	0.13	[0.09; 0.16]	6.8%	6.3%
Pan 2020	134	467	0.29	[0.25; 0.33]	8.5%	6.3%
Liu 2019	187	804	0.23	[0.20; 0.26]	14.7%	6.5%
Hu 2016	40	132	0.30	[0.23; 0.39]	2.4%	5.7%
Common effect model		2048	0.24	[0.22; 0.25]	37.5%	--
Random effects model			0.25	[0.19; 0.32]	--	36.1%
Heterogeneity: $I^2 = 89\%$, $\tau^2 = 0.0069$, $p < 0.01$						
period = 2016-2020						
Shen 2017	34	105	0.32	[0.24; 0.42]	1.9%	5.5%
Liu 2018	154	398	0.39	[0.34; 0.44]	7.3%	6.3%
Sun 2018	57	127	0.45	[0.36; 0.54]	2.3%	5.7%
Yu 2019	59	145	0.41	[0.33; 0.49]	2.7%	5.8%
Chen 2019	179	382	0.47	[0.42; 0.52]	7.0%	6.3%
Jiang 2021	330	1204	0.27	[0.25; 0.30]	22.0%	6.5%
Jiang 2020	103	553	0.19	[0.15; 0.22]	10.1%	6.4%
Luo 2020	17	37	0.46	[0.29; 0.63]	0.7%	4.2%
Luo 2020	87	207	0.42	[0.35; 0.49]	3.8%	6.0%
Common effect model		3158	0.32	[0.30; 0.33]	57.8%	--
Random effects model			0.37	[0.29; 0.44]	--	52.5%
Heterogeneity: $I^2 = 94\%$, $\tau^2 = 0.0127$, $p < 0.01$						
**Common effect model**		**5463**	**0.29**	**[0.28; 0.30]**	**100.0%**	**--**
**Random effects model**			**0.32**	**[0.27; 0.37]**	**--**	**100.0%**
Heterogeneity: $I^2 = 93\%$, $\tau^2 = 0.0111$, $p < 0.01$						

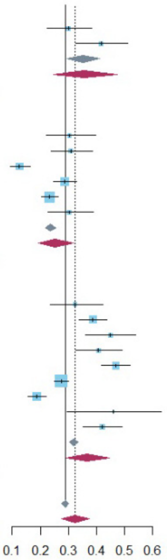

Figure S13

Amoxicillin [By period]

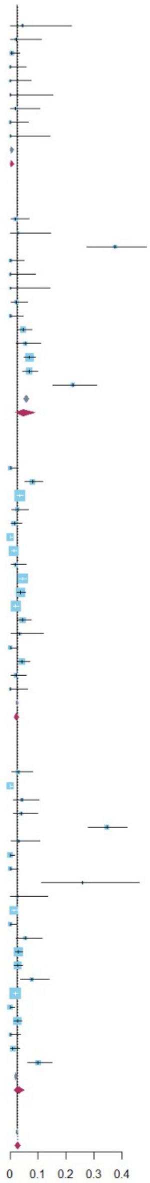

Study	Events	Total	Proportion	95%-CI	Weight (common)	Weight (random)
period = before 2005						
Wang 2019	1	23	0.04	[0.00; 0.22]	0.2%	1.0%
gao 2010	1	47	0.02	[0.00; 0.11]	0.3%	1.3%
Zhang 2002	1	164	0.01	[0.00; 0.03]	1.1%	1.8%
gao 2010	0	63	0.00	[0.00; 0.06]	0.4%	1.4%
Ruan 2007	0	47	0.00	[0.00; 0.08]	0.3%	1.3%
gao 2010	0	22	0.00	[0.00; 0.15]	0.1%	0.9%
Wang 2019	1	50	0.02	[0.00; 0.11]	0.3%	1.3%
Ruan 2007	0	54	0.00	[0.00; 0.07]	0.4%	1.4%
gao 2010	0	24	0.00	[0.00; 0.14]	0.2%	1.0%
Common effect model		494	0.00	[0.00; 0.01]	3.2%	--
Random effects model			0.00	[0.00; 0.01]	--	11.4%
Heterogeneity: $I^2 = 0\%$, $\tau^2 = 0$, $p = 0.78$						
period = 2006-2010						
Ruan 2007	2	102	0.02	[0.00; 0.07]	0.7%	1.6%
Gu 2007	1	36	0.03	[0.00; 0.15]	0.2%	1.2%
Cao 2007	32	85	0.38	[0.27; 0.49]	0.6%	1.6%
gao 2010	0	71	0.00	[0.00; 0.05]	0.5%	1.5%
gao 2010	0	39	0.00	[0.00; 0.09]	0.3%	1.2%
gao 2010	0	24	0.00	[0.00; 0.14]	0.2%	1.0%
Lin 2009	3	137	0.02	[0.00; 0.06]	0.9%	1.7%
Zheng 2010	0	77	0.00	[0.00; 0.05]	0.5%	1.5%
Zhou 2014	13	280	0.05	[0.02; 0.08]	1.8%	1.9%
Qi 2021	7	128	0.05	[0.02; 0.11]	0.8%	1.7%
Song 2014	41	600	0.07	[0.05; 0.09]	3.9%	2.0%
Zhang 2015	25	371	0.07	[0.04; 0.10]	2.4%	1.9%
Tan 2011	27	120	0.22	[0.15; 0.31]	0.8%	1.7%
Common effect model		2070	0.06	[0.05; 0.07]	13.4%	--
Random effects model			0.05	[0.02; 0.09]	--	20.3%
Heterogeneity: $I^2 = 91\%$, $\tau^2 = 0.0163$, $p < 0.01$						
period = 2011-2015						
Xu 2012	0	120	0.00	[0.00; 0.03]	0.8%	1.7%
Luo 2013	24	300	0.08	[0.05; 0.12]	1.9%	1.9%
Liu 2018	38	1117	0.03	[0.02; 0.05]	7.2%	2.0%
Meng 2016	4	155	0.03	[0.01; 0.06]	1.0%	1.7%
Zhang 2015	3	200	0.01	[0.00; 0.04]	1.3%	1.8%
Pan 2020	0	467	0.00	[0.00; 0.01]	3.0%	1.9%
Liu 2019	10	804	0.01	[0.01; 0.02]	5.2%	2.0%
Zhou 2018	3	150	0.02	[0.00; 0.06]	1.0%	1.7%
Zhang 2015	42	950	0.04	[0.03; 0.06]	6.1%	2.0%
Zhang 2015	26	700	0.04	[0.02; 0.05]	4.5%	2.0%
Zhou 2016	19	950	0.02	[0.01; 0.03]	6.1%	2.0%
Xie 2018	13	288	0.05	[0.02; 0.08]	1.9%	1.9%
Song 2016	2	58	0.03	[0.00; 0.12]	0.4%	1.4%
Hu 2016	0	132	0.00	[0.00; 0.03]	0.9%	1.7%
Fu 2017	14	324	0.04	[0.02; 0.07]	2.1%	1.9%
Song 2016	3	147	0.02	[0.00; 0.06]	1.0%	1.7%
ma 2017	0	56	0.00	[0.00; 0.06]	0.4%	1.4%
Common effect model		6918	0.02	[0.02; 0.03]	44.7%	--
Random effects model			0.02	[0.01; 0.03]	--	30.6%
Heterogeneity: $I^2 = 85\%$, $\tau^2 = 0.0035$, $p < 0.01$						
period = 2016-2020						
Shen 2017	3	105	0.03	[0.01; 0.08]	0.7%	1.6%
Liu 2018	0	398	0.00	[0.00; 0.01]	2.6%	1.9%
Suo 2019	4	96	0.04	[0.01; 0.10]	0.6%	1.6%
Suo 2020	4	100	0.04	[0.01; 0.10]	0.6%	1.6%
Zhang 2020	68	196	0.35	[0.28; 0.42]	1.3%	1.8%
Song 2019	2	65	0.03	[0.00; 0.11]	0.4%	1.5%
Yang 2019	0	232	0.00	[0.00; 0.02]	1.5%	1.8%
Sun 2018	0	127	0.00	[0.00; 0.03]	0.8%	1.7%
Wang 2019	7	27	0.26	[0.11; 0.46]	0.2%	1.0%
Wang 2018	1	39	0.03	[0.00; 0.13]	0.3%	1.2%
Lv 2020	7	557	0.01	[0.01; 0.03]	3.6%	1.9%
Yu 2019	0	145	0.00	[0.00; 0.03]	0.9%	1.7%
Gao 2020	6	111	0.05	[0.02; 0.11]	0.7%	1.6%
Song 2020	19	650	0.03	[0.02; 0.05]	4.2%	2.0%
Cui 2021	13	506	0.03	[0.01; 0.04]	3.3%	1.9%
Tang 2020	9	117	0.08	[0.04; 0.14]	0.8%	1.7%
Jiang 2021	22	1204	0.02	[0.01; 0.03]	7.8%	2.0%
Zhang 2021	0	231	0.00	[0.00; 0.02]	1.5%	1.8%
Jiang 2020	14	553	0.03	[0.01; 0.04]	3.6%	1.9%
Xu 2020	0	100	0.00	[0.00; 0.04]	0.6%	1.6%
Luo 2020	2	207	0.01	[0.00; 0.03]	1.3%	1.8%
He 2021	20	200	0.10	[0.06; 0.15]	1.3%	1.8%
Common effect model		5966	0.02	[0.02; 0.02]	38.6%	--
Random effects model			0.03	[0.01; 0.05]	--	37.6%
Heterogeneity: $I^2 = 93\%$, $\tau^2 = 0.0135$, $p < 0.01$						
**Common effect model**		**15448**	**0.02**	**[0.02; 0.03]**	**100.0%**	**--**
**Random effects model**			**0.03**	**[0.02; 0.04]**	**--**	**100.0%**
Heterogeneity: $I^2 = 90\%$, $\tau^2 = 0.0094$, $p < 0.01$						

Figure S14

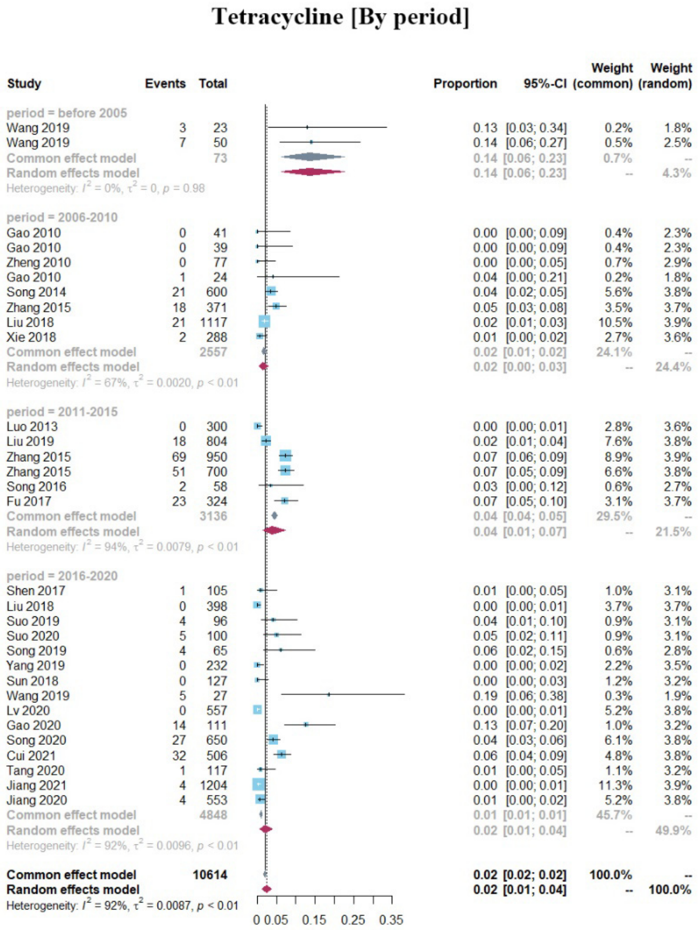
Tetracycline [By period]
Study Events Total Proportion 95%-CI Weight (common) Weight (random)
period = before 2005
Wang 2019 3 23 0.13 [0.03; 0.34] 0.2% 1.8%
Wang 2019 7 50 0.14 [0.06; 0.27] 0.5% 2.5%
Common effect model 73 0.14 [0.06; 0.23] 0.7% --
Random effects model 0.14 [0.06; 0.23] -- 4.3%
Heterogeneity: I^2 = 0%, τ^2 = 0, p = 0.98
period = 2006-2010
Gao 2010 0 41 0.00 [0.00; 0.09] 0.4% 2.3%
Gao 2010 0 39 0.00 [0.00; 0.09] 0.4% 2.3%
Zheng 2010 0 77 0.00 [0.00; 0.05] 0.7% 2.9%
Gao 2010 1 24 0.04 [0.00; 0.21] 0.2% 1.8%
Song 2014 21 600 0.04 [0.02; 0.05] 5.6% 3.8%
Zhang 2015 18 371 0.05 [0.03; 0.08] 3.5% 3.7%
Liu 2018 21 1117 0.02 [0.01; 0.03] 10.5% 3.9%
Xie 2018 2 288 0.01 [0.00; 0.02] 2.7% 3.6%
Common effect model 2557 0.02 [0.01; 0.02] 24.1% --
Random effects model 0.02 [0.00; 0.03] -- 24.4%
Heterogeneity: I^2 = 67%, τ^2 = 0.0020, p < 0.01
period = 2011-2015
Luo 2013 0 300 0.00 [0.00; 0.01] 2.8% 3.6%
Liu 2019 18 804 0.02 [0.01; 0.04] 7.6% 3.8%
Zhang 2015 69 950 0.07 [0.06; 0.09] 8.9% 3.9%
Zhang 2015 51 700 0.07 [0.05; 0.09] 6.6% 3.8%
Song 2016 2 58 0.03 [0.00; 0.12] 0.6% 2.7%
Fu 2017 23 324 0.07 [0.05; 0.10] 3.1% 3.7%
Common effect model 3136 0.04 [0.04; 0.05] 29.5% --
Random effects model 0.04 [0.01; 0.07] -- 21.5%
Heterogeneity: I^2 = 94%, τ^2 = 0.0079, p < 0.01
period = 2016-2020
Shen 2017 1 105 0.01 [0.00; 0.05] 1.0% 3.1%
Liu 2018 0 398 0.00 [0.00; 0.01] 3.7% 3.7%
Suo 2019 4 96 0.04 [0.01; 0.10] 0.9% 3.1%
Suo 2020 5 100 0.05 [0.02; 0.11] 0.9% 3.1%
Song 2019 4 65 0.06 [0.02; 0.15] 0.6% 2.8%
Yang 2019 0 232 0.00 [0.00; 0.02] 2.2% 3.5%
Sun 2018 0 127 0.00 [0.00; 0.03] 1.2% 3.2%
Wang 2019 5 27 0.19 [0.06; 0.38] 0.3% 1.9%
Lv 2020 0 557 0.00 [0.00; 0.01] 5.2% 3.8%
Gao 2020 14 111 0.13 [0.07; 0.20] 1.0% 3.2%
Song 2020 27 650 0.04 [0.03; 0.06] 6.1% 3.8%
Cui 2021 32 506 0.06 [0.04; 0.09] 4.8% 3.8%
Tang 2020 1 117 0.01 [0.00; 0.05] 1.1% 3.2%
Jiang 2021 4 1204 0.00 [0.00; 0.01] 11.3% 3.9%
Jiang 2020 4 553 0.01 [0.00; 0.02] 5.2% 3.8%
Common effect model 4848 0.01 [0.01; 0.01] 45.7% --
Random effects model 0.02 [0.01; 0.04] -- 49.9%
Heterogeneity: I^2 = 92%, τ^2 = 0.0096, p < 0.01
Common effect model 10614 0.02 [0.02; 0.02] 100.0% --
Random effects model 0.02 [0.01; 0.04] -- 100.0%
Heterogeneity: I^2 = 92%, τ^2 = 0.0087, p < 0.01
0 0.05 0.15 0.25 0.35

Figure S15

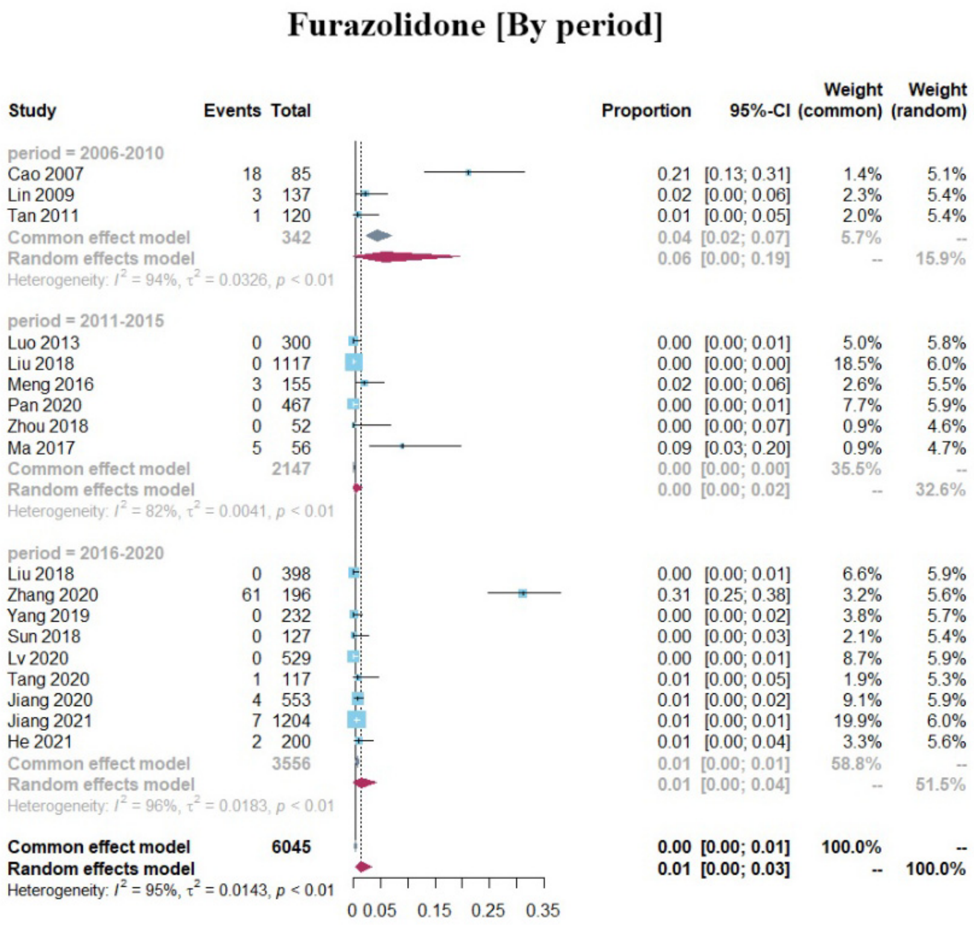
Furazolidone [By period]
Study Events Total Proportion 95%-CI Weight (common) Weight (random)
period = 2006-2010
Cao 2007 18 85 0.21 [0.13; 0.31] 1.4% 5.1%
Lin 2009 3 137 0.02 [0.00; 0.06] 2.3% 5.4%
Tan 2011 1 120 0.01 [0.00; 0.05] 2.0% 5.4%
Common effect model 342 0.04 [0.02; 0.07] 5.7% --
Random effects model 0.06 [0.00; 0.19] -- 15.9%
Heterogeneity: I^2 = 94%, τ^2 = 0.0326, p < 0.01
period = 2011-2015
Luo 2013 0 300 0.00 [0.00; 0.01] 5.0% 5.8%
Liu 2018 0 1117 0.00 [0.00; 0.00] 18.5% 6.0%
Meng 2016 3 155 0.02 [0.00; 0.06] 2.6% 5.5%
Pan 2020 0 467 0.00 [0.00; 0.01] 7.7% 5.9%
Zhou 2018 0 52 0.00 [0.00; 0.07] 0.9% 4.6%
Ma 2017 5 56 0.09 [0.03; 0.20] 0.9% 4.7%
Common effect model 2147 0.00 [0.00; 0.00] 35.5% --
Random effects model 0.00 [0.00; 0.02] -- 32.6%
Heterogeneity: I^2 = 82%, τ^2 = 0.0041, p < 0.01
period = 2016-2020
Liu 2018 0 398 0.00 [0.00; 0.01] 6.6% 5.9%
Zhang 2020 61 196 0.31 [0.25; 0.38] 3.2% 5.6%
Yang 2019 0 232 0.00 [0.00; 0.02] 3.8% 5.7%
Sun 2018 0 127 0.00 [0.00; 0.03] 2.1% 5.4%
Lv 2020 0 529 0.00 [0.00; 0.01] 8.7% 5.9%
Tang 2020 1 117 0.01 [0.00; 0.05] 1.9% 5.3%
Jiang 2020 4 553 0.01 [0.00; 0.02] 9.1% 5.9%
Jiang 2021 7 1204 0.01 [0.00; 0.01] 19.9% 6.0%
He 2021 2 200 0.01 [0.00; 0.04] 3.3% 5.6%
Common effect model 3556 0.01 [0.00; 0.01] 58.8% --
Random effects model 0.01 [0.00; 0.04] -- 51.5%
Heterogeneity: I^2 = 96%, τ^2 = 0.0183, p < 0.01
Common effect model 6045 0.00 [0.00; 0.01] 100.0% --
Random effects model 0.01 [0.00; 0.03] -- 100.0%
Heterogeneity: I^2 = 95%, τ^2 = 0.0143, p < 0.01
0 0.05 0.15 0.25 0.35

Figure S16

Clarithromycin [By gender]

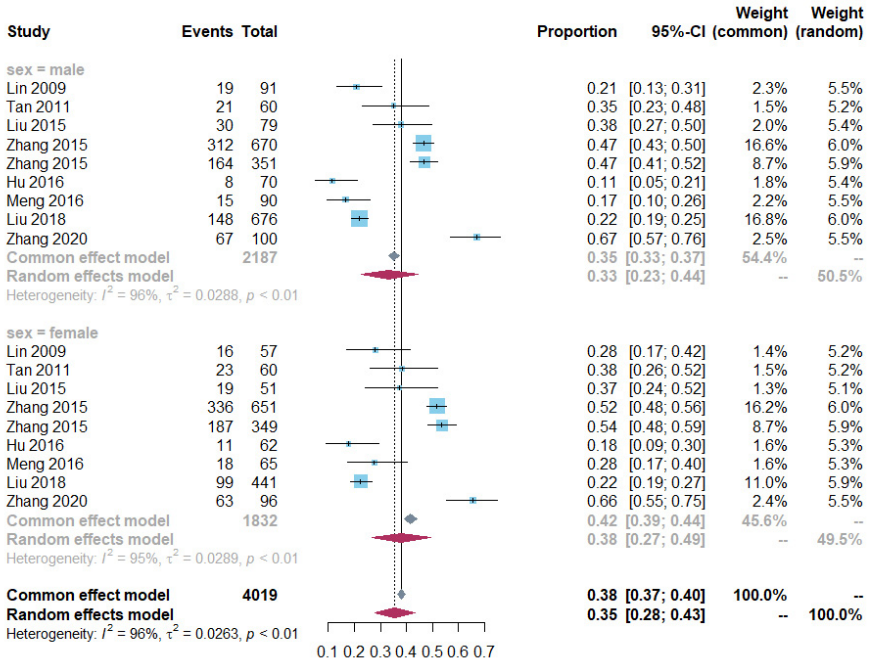

Figure S17

Levofloxacin [By gender]

Study	Events	Total	Proportion	95%-CI	Weight (common)	Weight (random)
sex = male						
Lin 2009	28	91	0.31	[0.22; 0.41]	2.3%	6.2%
Tan 2011	27	60	0.45	[0.32; 0.58]	1.5%	5.9%
Zhang 2015	292	670	0.44	[0.40; 0.47]	16.9%	6.6%
Zhang 2015	174	351	0.50	[0.44; 0.55]	8.9%	6.5%
Hu 2016	16	70	0.23	[0.14; 0.34]	1.8%	6.0%
Meng 2016	3	90	0.03	[0.01; 0.09]	2.3%	6.2%
Liu 2018	114	676	0.17	[0.14; 0.20]	17.1%	6.6%
Zhang 2020	42	100	0.42	[0.32; 0.52]	2.5%	6.2%
Common effect model		2108	0.32	[0.30; 0.34]	53.4%	--
Random effects model			0.30	[0.18; 0.43]	--	50.1%
Heterogeneity: $I^2 = 97\%$, $\tau^2 = 0.0362$, $p < 0.01$						
sex = female						
Lin 2009	21	57	0.37	[0.24; 0.51]	1.5%	5.9%
Tan 2011	23	120	0.19	[0.13; 0.27]	3.0%	6.3%
Zhang 2015	357	651	0.55	[0.51; 0.59]	16.5%	6.6%
Zhang 2015	207	349	0.59	[0.54; 0.65]	8.8%	6.5%
Hu 2016	24	62	0.39	[0.27; 0.52]	1.6%	6.0%
Meng 2016	6	65	0.09	[0.03; 0.19]	1.7%	6.0%
Liu 2018	100	441	0.23	[0.19; 0.27]	11.2%	6.5%
Zhang 2020	39	96	0.41	[0.31; 0.51]	2.4%	6.2%
Common effect model		1841	0.41	[0.39; 0.44]	46.6%	--
Random effects model			0.34	[0.22; 0.48]	--	49.9%
Heterogeneity: $I^2 = 97\%$, $\tau^2 = 0.0394$, $p < 0.01$						
**Common effect model**		**3949**	**0.36**	**[0.35; 0.38]**	**100.0%**	**--**
**Random effects model**			**0.32**	**[0.24; 0.42]**	**--**	**100.0%**
Heterogeneity: $I^2 = 97\%$, $\tau^2 = 0.0355$, $p < 0.01$						

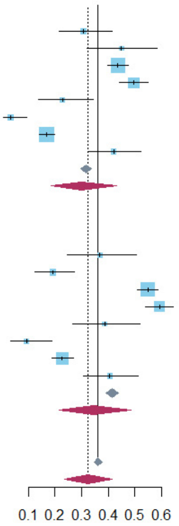

Figure S18

Metronidazole [By gender]

Study	Events	Total	Proportion	95%-CI	Weight (common)	Weight (random)
**sex = male**						
Zhang 2002	48	91	0.53	[0.42; 0.63]	2.2%	4.8%
Hu 2004	11	22	0.50	[0.28; 0.72]	0.5%	3.6%
Gu 2007	6	15	0.40	[0.16; 0.68]	0.4%	3.1%
Lin 2009	67	91	0.74	[0.63; 0.82]	2.2%	4.8%
Tan 2011	47	60	0.78	[0.66; 0.88]	1.5%	4.5%
Zhang 2015	398	670	0.59	[0.56; 0.63]	16.2%	5.2%
Zhang 2015	209	351	0.60	[0.54; 0.65]	8.5%	5.2%
Hu 2016	45	70	0.64	[0.52; 0.75]	1.7%	4.6%
Meng 2016	84	90	0.93	[0.86; 0.98]	2.2%	4.8%
Liu 2018	517	676	0.76	[0.73; 0.80]	16.4%	5.2%
Zhang 2020	98	100	0.98	[0.93; 1.00]	2.4%	4.8%
Common effect model		2236	0.70	[0.68; 0.72]	54.2%	--
Random effects model			0.71	[0.61; 0.80]	--	50.6%
Heterogeneity: $I^2 = 95\%$, $\tau^2 = 0.0263$, $p < 0.01$						
**sex = female**						
Zhang 2002	37	73	0.51	[0.39; 0.63]	1.8%	4.6%
Hu 2004	10	11	0.91	[0.59; 1.00]	0.3%	2.8%
Gu 2007	10	21	0.48	[0.26; 0.70]	0.5%	3.6%
Lin 2009	50	57	0.88	[0.76; 0.95]	1.4%	4.5%
Tan 2011	52	60	0.87	[0.75; 0.94]	1.5%	4.5%
Zhang 2015	452	651	0.69	[0.66; 0.73]	15.8%	5.2%
Zhang 2015	238	349	0.68	[0.63; 0.73]	8.5%	5.2%
Hu 2016	40	62	0.65	[0.51; 0.76]	1.5%	4.5%
Meng 2016	62	65	0.95	[0.87; 0.99]	1.6%	4.6%
Liu 2018	357	441	0.81	[0.77; 0.85]	10.7%	5.2%
Zhang 2020	95	96	0.99	[0.94; 1.00]	2.3%	4.8%
Common effect model		1886	0.76	[0.74; 0.78]	45.8%	--
Random effects model			0.79	[0.70; 0.87]	--	49.4%
Heterogeneity: $I^2 = 93\%$, $\tau^2 = 0.0240$, $p < 0.01$						
**Common effect model**		**4122**	**0.73**	**[0.71; 0.74]**	**100.0%**	**--**
**Random effects model**			**0.75**	**[0.69; 0.81]**	**--**	**100.0%**
Heterogeneity: $I^2 = 94\%$, $\tau^2 = 0.0234$, $p < 0.01$						

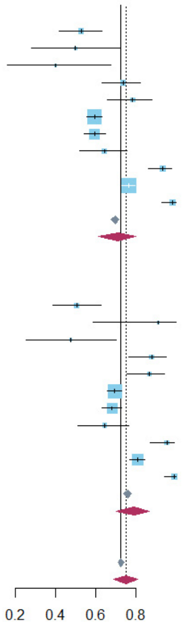

Figure S19

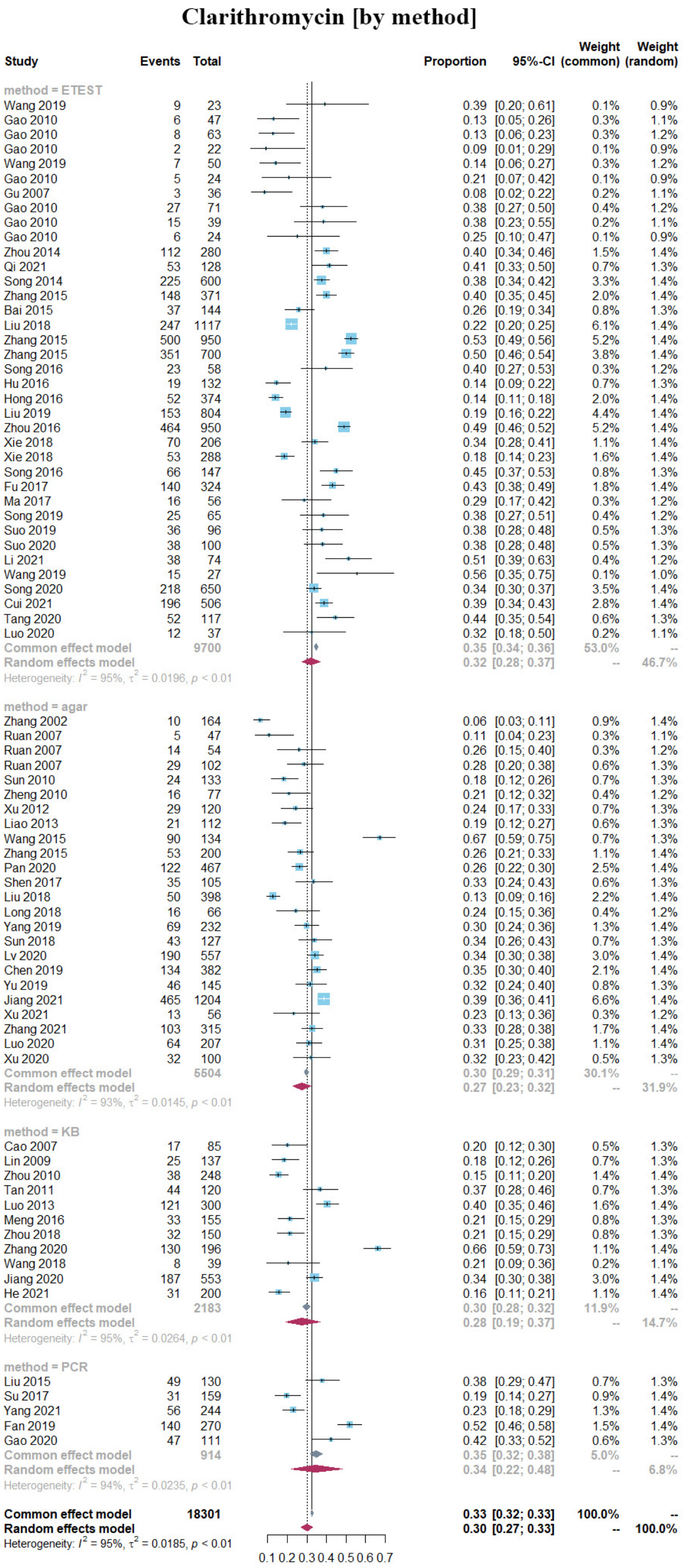

Clarithromycin [by method]
Study Events Total Proportion 95%-CI Weight (common) Weight (random)
method = ETEST
Wang 2019 9 23 0.39 [0.20; 0.61] 0.1% 0.9%
Gao 2010 6 47 0.13 [0.05; 0.26] 0.3% 1.1%
Gao 2010 8 63 0.13 [0.06; 0.23] 0.3% 1.2%
Gao 2010 2 22 0.09 [0.01; 0.29] 0.1% 0.9%
Wang 2019 7 50 0.14 [0.06; 0.27] 0.3% 1.2%
Gao 2010 5 24 0.21 [0.07; 0.42] 0.1% 0.9%
Gu 2007 3 36 0.08 [0.02; 0.22] 0.2% 1.1%
Gao 2010 27 71 0.38 [0.27; 0.50] 0.4% 1.2%
Gao 2010 15 39 0.38 [0.23; 0.55] 0.2% 1.1%
Gao 2010 6 24 0.25 [0.10; 0.47] 0.1% 0.9%
Zhou 2014 112 280 0.40 [0.34; 0.46] 1.5% 1.4%
Qi 2021 53 128 0.41 [0.33; 0.50] 0.7% 1.3%
Song 2014 225 600 0.38 [0.34; 0.42] 3.3% 1.4%
Zhang 2015 148 371 0.40 [0.35; 0.45] 2.0% 1.4%
Bai 2015 37 144 0.26 [0.19; 0.34] 0.8% 1.3%
Liu 2018 247 1117 0.22 [0.20; 0.25] 6.1% 1.4%
Zhang 2015 500 950 0.53 [0.49; 0.56] 5.2% 1.4%
Zhang 2015 351 700 0.50 [0.46; 0.54] 3.8% 1.4%
Song 2016 23 58 0.40 [0.27; 0.53] 0.3% 1.2%
Hu 2016 19 132 0.14 [0.09; 0.22] 0.7% 1.3%
Hong 2016 52 374 0.14 [0.11; 0.18] 2.0% 1.4%
Liu 2019 153 804 0.19 [0.16; 0.22] 4.4% 1.4%
Zhou 2016 464 950 0.49 [0.46; 0.52] 5.2% 1.4%
Xie 2018 70 206 0.34 [0.28; 0.41] 1.1% 1.4%
Xie 2018 53 288 0.18 [0.14; 0.23] 1.6% 1.4%
Song 2016 66 147 0.45 [0.37; 0.53] 0.8% 1.3%
Fu 2017 140 324 0.43 [0.38; 0.49] 1.8% 1.4%
Ma 2017 16 56 0.29 [0.17; 0.42] 0.3% 1.2%
Song 2019 25 65 0.38 [0.27; 0.51] 0.4% 1.2%
Suo 2019 36 96 0.38 [0.28; 0.48] 0.5% 1.3%
Suo 2020 38 100 0.38 [0.28; 0.48] 0.5% 1.3%
Li 2021 38 74 0.51 [0.39; 0.63] 0.4% 1.2%
Wang 2019 15 27 0.56 [0.35; 0.75] 0.1% 1.0%
Song 2020 218 650 0.34 [0.30; 0.37] 3.5% 1.4%
Cui 2021 196 506 0.39 [0.34; 0.43] 2.8% 1.4%
Tang 2020 52 117 0.44 [0.35; 0.54] 0.6% 1.3%
Luo 2020 12 37 0.32 [0.18; 0.50] 0.2% 1.1%
Common effect model 9700 0.35 [0.34; 0.36] 53.0% --
Random effects model 0.32 [0.28; 0.37] -- 46.7%
Heterogeneity: I^2 = 95%, τ^2 = 0.0196, p < 0.01
method = agar
Zhang 2002 10 164 0.06 [0.03; 0.11] 0.9% 1.4%
Ruan 2007 5 47 0.11 [0.04; 0.23] 0.3% 1.1%
Ruan 2007 14 54 0.26 [0.15; 0.40] 0.3% 1.2%
Ruan 2007 29 102 0.28 [0.20; 0.38] 0.6% 1.3%
Sun 2010 24 133 0.18 [0.12; 0.26] 0.7% 1.3%
Zheng 2010 16 77 0.21 [0.12; 0.32] 0.4% 1.2%
Xu 2012 29 120 0.24 [0.17; 0.33] 0.7% 1.3%
Liao 2013 21 112 0.19 [0.12; 0.27] 0.6% 1.3%
Wang 2015 90 134 0.67 [0.59; 0.75] 0.7% 1.3%
Zhang 2015 53 200 0.26 [0.21; 0.33] 1.1% 1.4%
Pan 2020 122 467 0.26 [0.22; 0.30] 2.5% 1.4%
Shen 2017 35 105 0.33 [0.24; 0.43] 0.6% 1.3%
Liu 2018 50 398 0.13 [0.09; 0.16] 2.2% 1.4%
Long 2018 16 66 0.24 [0.15; 0.36] 0.4% 1.2%
Yang 2019 69 232 0.30 [0.24; 0.36] 1.3% 1.4%
Sun 2018 43 127 0.34 [0.26; 0.43] 0.7% 1.3%
Lv 2020 190 557 0.34 [0.30; 0.38] 3.0% 1.4%
Chen 2019 134 382 0.35 [0.30; 0.40] 2.1% 1.4%
Yu 2019 46 145 0.32 [0.24; 0.40] 0.8% 1.3%
Jiang 2021 465 1204 0.39 [0.36; 0.41] 6.6% 1.4%
Xu 2021 13 56 0.23 [0.13; 0.36] 0.3% 1.2%
Zhang 2021 103 315 0.33 [0.28; 0.38] 1.7% 1.4%
Luo 2020 64 207 0.31 [0.25; 0.38] 1.1% 1.4%
Xu 2020 32 100 0.32 [0.23; 0.42] 0.5% 1.3%
Common effect model 5504 0.30 [0.29; 0.31] 30.1% --
Random effects model 0.27 [0.23; 0.32] -- 31.9%
Heterogeneity: I^2 = 93%, τ^2 = 0.0145, p < 0.01
method = KB
Cao 2007 17 85 0.20 [0.12; 0.30] 0.5% 1.3%
Lin 2009 25 137 0.18 [0.12; 0.26] 0.7% 1.3%
Zhou 2010 38 248 0.15 [0.11; 0.20] 1.4% 1.4%
Tan 2011 44 120 0.37 [0.28; 0.46] 0.7% 1.3%
Luo 2013 121 300 0.40 [0.35; 0.46] 1.6% 1.4%
Meng 2016 33 155 0.21 [0.15; 0.29] 0.8% 1.3%
Zhou 2018 32 150 0.21 [0.15; 0.29] 0.8% 1.3%
Zhang 2020 130 196 0.66 [0.59; 0.73] 1.1% 1.4%
Wang 2018 8 39 0.21 [0.09; 0.36] 0.2% 1.1%
Jiang 2020 187 553 0.34 [0.30; 0.38] 3.0% 1.4%
He 2021 31 200 0.16 [0.11; 0.21] 1.1% 1.4%
Common effect model 2183 0.30 [0.28; 0.32] 11.9% --
Random effects model 0.28 [0.19; 0.37] -- 14.7%
Heterogeneity: I^2 = 95%, τ^2 = 0.0264, p < 0.01
method = PCR
Liu 2015 49 130 0.38 [0.29; 0.47] 0.7% 1.3%
Su 2017 31 159 0.19 [0.14; 0.27] 0.9% 1.4%
Yang 2021 56 244 0.23 [0.18; 0.29] 1.3% 1.4%
Fan 2019 140 270 0.52 [0.46; 0.58] 1.5% 1.4%
Gao 2020 47 111 0.42 [0.33; 0.52] 0.6% 1.3%
Common effect model 914 0.35 [0.32; 0.38] 5.0% --
Random effects model 0.34 [0.22; 0.48] -- 6.8%
Heterogeneity: I^2 = 94%, τ^2 = 0.0235, p < 0.01
Common effect model 18301 0.33 [0.32; 0.33] 100.0% --
Random effects model 0.30 [0.27; 0.33] -- 100.0%
Heterogeneity: I^2 = 95%, τ^2 = 0.0185, p < 0.01
0.1 0.2 0.3 0.4 0.5 0.6 0.7

Figure S20

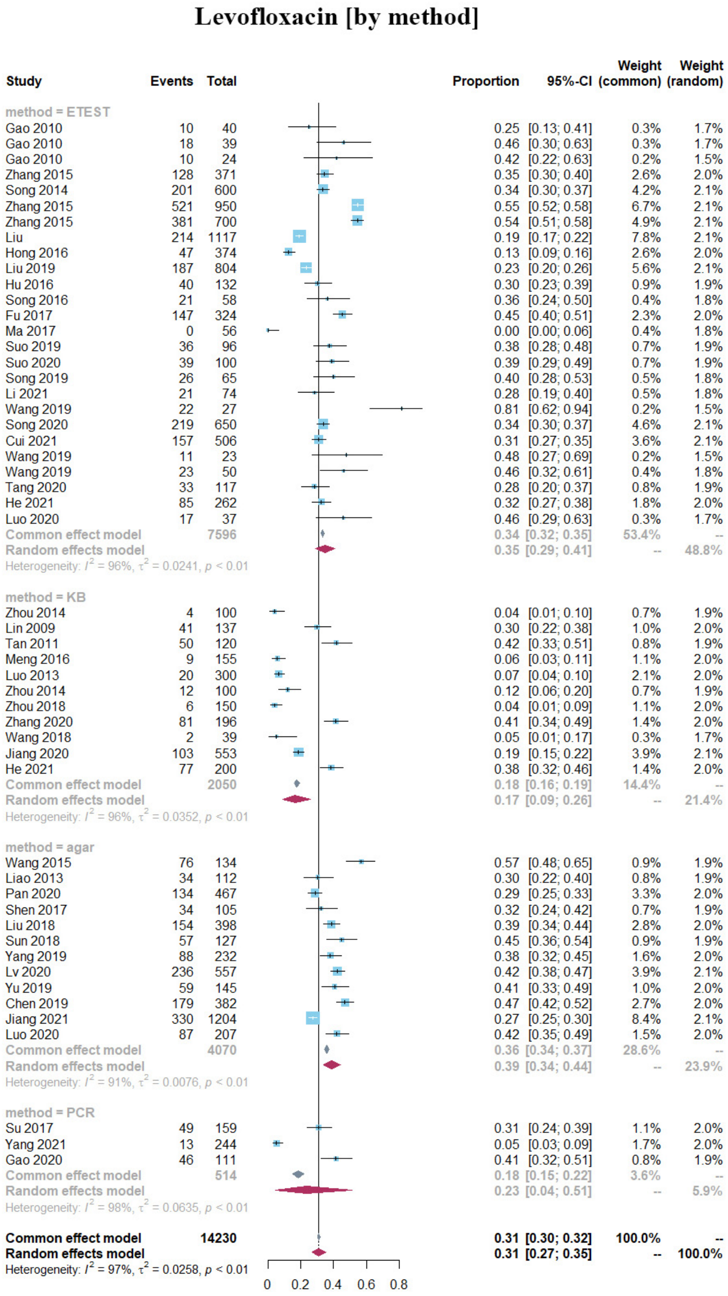
Levofloxacin [by method]
Study | Events | Total | Proportion | 95%-CI | Weight (common) | Weight (random)
method = ETEST
Gao 2010 10 40 0.25 [0.13; 0.41] 0.3% 1.7%
Gao 2010 18 39 0.46 [0.30; 0.63] 0.3% 1.7%
Gao 2010 10 24 0.42 [0.22; 0.63] 0.2% 1.5%
Zhang 2015 128 371 0.35 [0.30; 0.40] 2.6% 2.0%
Song 2014 201 600 0.34 [0.30; 0.37] 4.2% 2.1%
Zhang 2015 521 950 0.55 [0.52; 0.58] 6.7% 2.1%
Zhang 2015 381 700 0.54 [0.51; 0.58] 4.9% 2.1%
Liu 214 1117 0.19 [0.17; 0.22] 7.8% 2.1%
Hong 2016 47 374 0.13 [0.09; 0.16] 2.6% 2.0%
Liu 2019 187 804 0.23 [0.20; 0.26] 5.6% 2.1%
Hu 2016 40 132 0.30 [0.23; 0.39] 0.9% 1.9%
Song 2016 21 58 0.36 [0.24; 0.50] 0.4% 1.8%
Fu 2017 147 324 0.45 [0.40; 0.51] 2.3% 2.0%
Ma 2017 0 56 0.00 [0.00; 0.06] 0.4% 1.8%
Suo 2019 36 96 0.38 [0.28; 0.48] 0.7% 1.9%
Suo 2020 39 100 0.39 [0.29; 0.49] 0.7% 1.9%
Song 2019 26 65 0.40 [0.28; 0.53] 0.5% 1.8%
Li 2021 21 74 0.28 [0.19; 0.40] 0.5% 1.8%
Wang 2019 22 27 0.81 [0.62; 0.94] 0.2% 1.5%
Song 2020 219 650 0.34 [0.30; 0.37] 4.6% 2.1%
Cui 2021 157 506 0.31 [0.27; 0.35] 3.6% 2.1%
Wang 2019 11 23 0.48 [0.27; 0.69] 0.2% 1.5%
Wang 2019 23 50 0.46 [0.32; 0.61] 0.4% 1.8%
Tang 2020 33 117 0.28 [0.20; 0.37] 0.8% 1.9%
He 2021 85 262 0.32 [0.27; 0.38] 1.8% 2.0%
Luo 2020 17 37 0.46 [0.29; 0.63] 0.3% 1.7%
Common effect model 7596 0.34 [0.32; 0.35] 53.4% --
Random effects model 0.35 [0.29; 0.41] -- 48.8%
Heterogeneity: I^2 = 96%, τ^2 = 0.0241, p < 0.01
method = KB
Zhou 2014 4 100 0.04 [0.01; 0.10] 0.7% 1.9%
Lin 2009 41 137 0.30 [0.22; 0.38] 1.0% 2.0%
Tan 2011 50 120 0.42 [0.33; 0.51] 0.8% 1.9%
Meng 2016 9 155 0.06 [0.03; 0.11] 1.1% 2.0%
Luo 2013 20 300 0.07 [0.04; 0.10] 2.1% 2.0%
Zhou 2014 12 100 0.12 [0.06; 0.20] 0.7% 1.9%
Zhou 2018 6 150 0.04 [0.01; 0.09] 1.1% 2.0%
Zhang 2020 81 196 0.41 [0.34; 0.49] 1.4% 2.0%
Wang 2018 2 39 0.05 [0.01; 0.17] 0.3% 1.7%
Jiang 2020 103 553 0.19 [0.15; 0.22] 3.9% 2.1%
He 2021 77 200 0.38 [0.32; 0.46] 1.4% 2.0%
Common effect model 2050 0.18 [0.16; 0.19] 14.4% --
Random effects model 0.17 [0.09; 0.26] -- 21.4%
Heterogeneity: I^2 = 96%, τ^2 = 0.0352, p < 0.01
method = agar
Wang 2015 76 134 0.57 [0.48; 0.65] 0.9% 1.9%
Liao 2013 34 112 0.30 [0.22; 0.40] 0.8% 1.9%
Pan 2020 134 467 0.29 [0.25; 0.33] 3.3% 2.0%
Shen 2017 34 105 0.32 [0.24; 0.42] 0.7% 1.9%
Liu 2018 154 398 0.39 [0.34; 0.44] 2.8% 2.0%
Sun 2018 57 127 0.45 [0.36; 0.54] 0.9% 1.9%
Yang 2019 88 232 0.38 [0.32; 0.45] 1.6% 2.0%
Lv 2020 236 557 0.42 [0.38; 0.47] 3.9% 2.1%
Yu 2019 59 145 0.41 [0.33; 0.49] 1.0% 2.0%
Chen 2019 179 382 0.47 [0.42; 0.52] 2.7% 2.0%
Jiang 2021 330 1204 0.27 [0.25; 0.30] 8.4% 2.1%
Luo 2020 87 207 0.42 [0.35; 0.49] 1.5% 2.0%
Common effect model 4070 0.36 [0.34; 0.37] 28.6% --
Random effects model 0.39 [0.34; 0.44] -- 23.9%
Heterogeneity: I^2 = 91%, τ^2 = 0.0076, p < 0.01
method = PCR
Su 2017 49 159 0.31 [0.24; 0.39] 1.1% 2.0%
Yang 2021 13 244 0.05 [0.03; 0.09] 1.7% 2.0%
Gao 2020 46 111 0.41 [0.32; 0.51] 0.8% 1.9%
Common effect model 514 0.18 [0.15; 0.22] 3.6% --
Random effects model 0.23 [0.04; 0.51] -- 5.9%
Heterogeneity: I^2 = 98%, τ^2 = 0.0635, p < 0.01
Common effect model 14230 0.31 [0.30; 0.32] 100.0% --
Random effects model 0.31 [0.27; 0.35] -- 100.0%
Heterogeneity: I^2 = 97%, τ^2 = 0.0258, p < 0.01
0 0.2 0.4 0.6 0.8

Figure S21

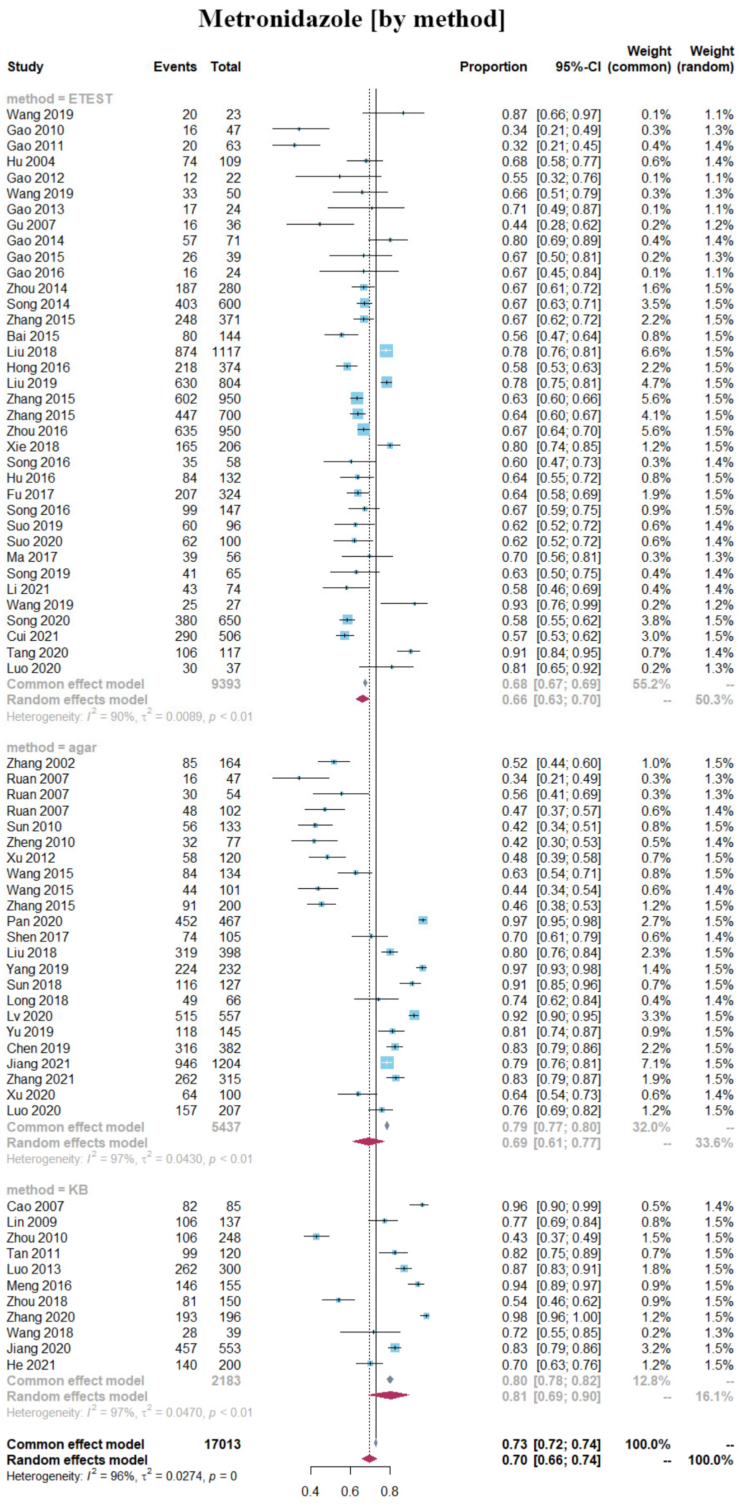

Metronidazole [by method]
Study Events Total Proportion 95%-CI Weight (common) Weight (random)
method = ETEST
Wang 2019 20 23 0.87 [0.66; 0.97] 0.1% 1.1%
Gao 2010 16 47 0.34 [0.21; 0.49] 0.3% 1.3%
Gao 2011 20 63 0.32 [0.21; 0.45] 0.4% 1.4%
Hu 2004 74 109 0.68 [0.58; 0.77] 0.6% 1.4%
Gao 2012 12 22 0.55 [0.32; 0.76] 0.1% 1.1%
Wang 2019 33 50 0.66 [0.51; 0.79] 0.3% 1.3%
Gao 2013 17 24 0.71 [0.49; 0.87] 0.1% 1.1%
Gu 2007 16 36 0.44 [0.28; 0.62] 0.2% 1.2%
Gao 2014 57 71 0.80 [0.69; 0.89] 0.4% 1.4%
Gao 2015 26 39 0.67 [0.50; 0.81] 0.2% 1.3%
Gao 2016 16 24 0.67 [0.45; 0.84] 0.1% 1.1%
Zhou 2014 187 280 0.67 [0.61; 0.72] 1.6% 1.5%
Song 2014 403 600 0.67 [0.63; 0.71] 3.5% 1.5%
Zhang 2015 248 371 0.67 [0.62; 0.72] 2.2% 1.5%
Bai 2015 80 144 0.56 [0.47; 0.64] 0.8% 1.5%
Liu 2018 874 1117 0.78 [0.76; 0.81] 6.6% 1.5%
Hong 2016 218 374 0.58 [0.53; 0.63] 2.2% 1.5%
Liu 2019 630 804 0.78 [0.75; 0.81] 4.7% 1.5%
Zhang 2015 602 950 0.63 [0.60; 0.66] 5.6% 1.5%
Zhang 2015 447 700 0.64 [0.60; 0.67] 4.1% 1.5%
Zhou 2016 635 950 0.67 [0.64; 0.70] 5.6% 1.5%
Xie 2018 165 206 0.80 [0.74; 0.85] 1.2% 1.5%
Song 2016 35 58 0.60 [0.47; 0.73] 0.3% 1.4%
Hu 2016 84 132 0.64 [0.55; 0.72] 0.8% 1.5%
Fu 2017 207 324 0.64 [0.58; 0.69] 1.9% 1.5%
Song 2016 99 147 0.67 [0.59; 0.75] 0.9% 1.5%
Suo 2019 60 96 0.62 [0.52; 0.72] 0.6% 1.4%
Suo 2020 62 100 0.62 [0.52; 0.72] 0.6% 1.4%
Ma 2017 39 56 0.70 [0.56; 0.81] 0.3% 1.3%
Song 2019 41 65 0.63 [0.50; 0.75] 0.4% 1.4%
Li 2021 43 74 0.58 [0.46; 0.69] 0.4% 1.4%
Wang 2019 25 27 0.93 [0.76; 0.99] 0.2% 1.2%
Song 2020 380 650 0.58 [0.55; 0.62] 3.8% 1.5%
Cui 2021 290 506 0.57 [0.53; 0.62] 3.0% 1.5%
Tang 2020 106 117 0.91 [0.84; 0.95] 0.7% 1.4%
Luo 2020 30 37 0.81 [0.65; 0.92] 0.2% 1.3%
Common effect model 9393 0.68 [0.67; 0.69] 55.2% --
Random effects model 0.66 [0.63; 0.70] -- 50.3%
Heterogeneity: I^2 = 90%, τ^2 = 0.0089, p < 0.01
method = agar
Zhang 2002 85 164 0.52 [0.44; 0.60] 1.0% 1.5%
Ruan 2007 16 47 0.34 [0.21; 0.49] 0.3% 1.3%
Ruan 2007 30 54 0.56 [0.41; 0.69] 0.3% 1.3%
Ruan 2007 48 102 0.47 [0.37; 0.57] 0.6% 1.4%
Sun 2010 56 133 0.42 [0.34; 0.51] 0.8% 1.5%
Zheng 2010 32 77 0.42 [0.30; 0.53] 0.5% 1.4%
Xu 2012 58 120 0.48 [0.39; 0.58] 0.7% 1.5%
Wang 2015 84 134 0.63 [0.54; 0.71] 0.8% 1.5%
Wang 2015 44 101 0.44 [0.34; 0.54] 0.6% 1.4%
Zhang 2015 91 200 0.46 [0.38; 0.53] 1.2% 1.5%
Pan 2020 452 467 0.97 [0.95; 0.98] 2.7% 1.5%
Shen 2017 74 105 0.70 [0.61; 0.79] 0.6% 1.4%
Liu 2018 319 398 0.80 [0.76; 0.84] 2.3% 1.5%
Yang 2019 224 232 0.97 [0.93; 0.98] 1.4% 1.5%
Sun 2018 116 127 0.91 [0.85; 0.96] 0.7% 1.5%
Long 2018 49 66 0.74 [0.62; 0.84] 0.4% 1.4%
Lv 2020 515 557 0.92 [0.90; 0.95] 3.3% 1.5%
Yu 2019 118 145 0.81 [0.74; 0.87] 0.9% 1.5%
Chen 2019 316 382 0.83 [0.79; 0.86] 2.2% 1.5%
Jiang 2021 946 1204 0.79 [0.76; 0.81] 7.1% 1.5%
Zhang 2021 262 315 0.83 [0.79; 0.87] 1.9% 1.5%
Xu 2020 64 100 0.64 [0.54; 0.73] 0.6% 1.4%
Luo 2020 157 207 0.76 [0.69; 0.82] 1.2% 1.5%
Common effect model 5437 0.79 [0.77; 0.80] 32.0% --
Random effects model 0.69 [0.61; 0.77] -- 33.6%
Heterogeneity: I^2 = 97%, τ^2 = 0.0430, p < 0.01
method = KB
Cao 2007 82 85 0.96 [0.90; 0.99] 0.5% 1.4%
Lin 2009 106 137 0.77 [0.69; 0.84] 0.8% 1.5%
Zhou 2010 106 248 0.43 [0.37; 0.49] 1.5% 1.5%
Tan 2011 99 120 0.82 [0.75; 0.89] 0.7% 1.5%
Luo 2013 262 300 0.87 [0.83; 0.91] 1.8% 1.5%
Meng 2016 146 155 0.94 [0.89; 0.97] 0.9% 1.5%
Zhou 2018 81 150 0.54 [0.46; 0.62] 0.9% 1.5%
Zhang 2020 193 196 0.98 [0.96; 1.00] 1.2% 1.5%
Wang 2018 28 39 0.72 [0.55; 0.85] 0.2% 1.3%
Jiang 2020 457 553 0.83 [0.79; 0.86] 3.2% 1.5%
He 2021 140 200 0.70 [0.63; 0.76] 1.2% 1.5%
Common effect model 2183 0.80 [0.78; 0.82] 12.8% --
Random effects model 0.81 [0.69; 0.90] -- 16.1%
Heterogeneity: I^2 = 97%, τ^2 = 0.0470, p < 0.01
Common effect model 17013 0.73 [0.72; 0.74] 100.0% --
Random effects model 0.70 [0.66; 0.74] -- 100.0%
Heterogeneity: I^2 = 96%, τ^2 = 0.0274, p = 0
0.4 0.6 0.8

Figure S22

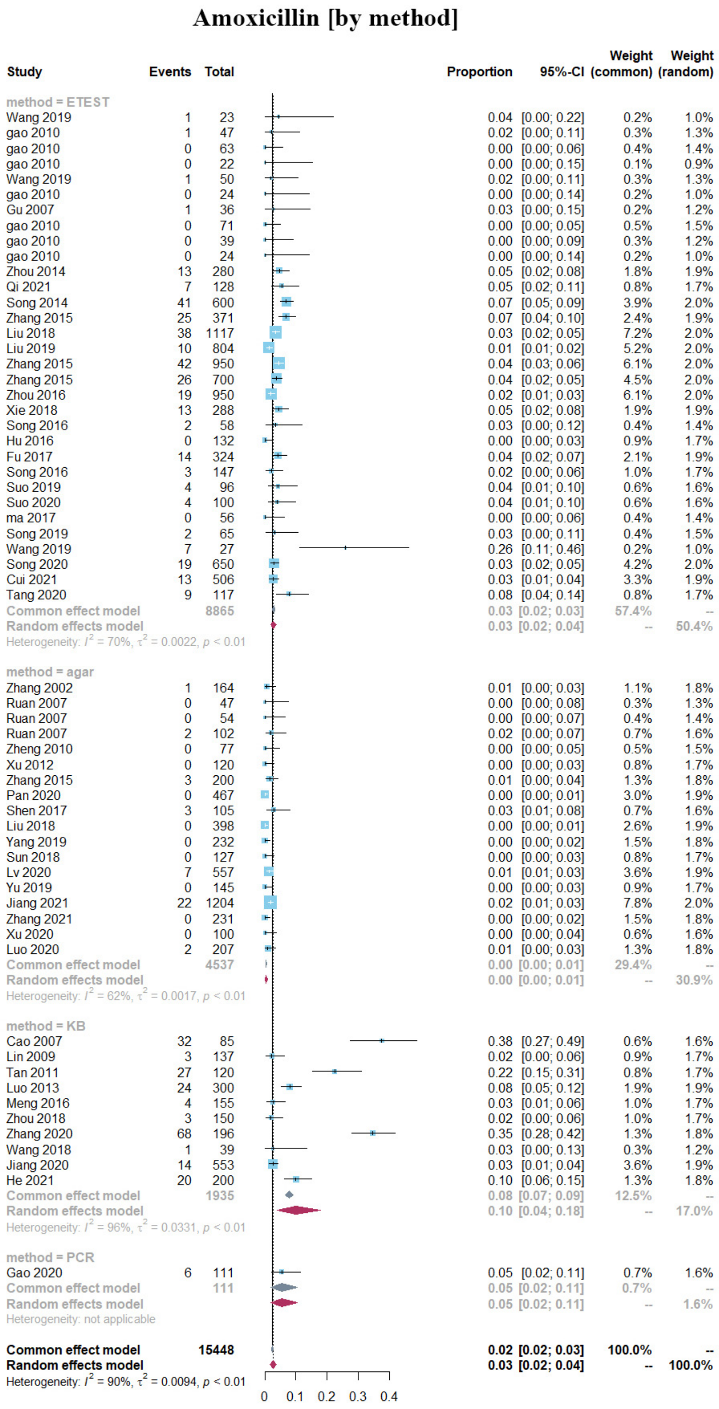

Amoxicillin [by method]
Study Events Total Proportion 95%-CI Weight (common) Weight (random)
method = ETEST
Wang 2019 1 23 0.04 [0.00; 0.22] 0.2% 1.0%
gao 2010 1 47 0.02 [0.00; 0.11] 0.3% 1.3%
gao 2010 0 63 0.00 [0.00; 0.06] 0.4% 1.4%
gao 2010 0 22 0.00 [0.00; 0.15] 0.1% 0.9%
Wang 2019 1 50 0.02 [0.00; 0.11] 0.3% 1.3%
gao 2010 0 24 0.00 [0.00; 0.14] 0.2% 1.0%
Gu 2007 1 36 0.03 [0.00; 0.15] 0.2% 1.2%
gao 2010 0 71 0.00 [0.00; 0.05] 0.5% 1.5%
gao 2010 0 39 0.00 [0.00; 0.09] 0.3% 1.2%
gao 2010 0 24 0.00 [0.00; 0.14] 0.2% 1.0%
Zhou 2014 13 280 0.05 [0.02; 0.08] 1.8% 1.9%
Qi 2021 7 128 0.05 [0.02; 0.11] 0.8% 1.7%
Song 2014 41 600 0.07 [0.05; 0.09] 3.9% 2.0%
Zhang 2015 25 371 0.07 [0.04; 0.10] 2.4% 1.9%
Liu 2018 38 1117 0.03 [0.02; 0.05] 7.2% 2.0%
Liu 2019 10 804 0.01 [0.01; 0.02] 5.2% 2.0%
Zhang 2015 42 950 0.04 [0.03; 0.06] 6.1% 2.0%
Zhang 2015 26 700 0.04 [0.02; 0.05] 4.5% 2.0%
Zhou 2016 19 950 0.02 [0.01; 0.03] 6.1% 2.0%
Xie 2018 13 288 0.05 [0.02; 0.08] 1.9% 1.9%
Song 2016 2 58 0.03 [0.00; 0.12] 0.4% 1.4%
Hu 2016 0 132 0.00 [0.00; 0.03] 0.9% 1.7%
Fu 2017 14 324 0.04 [0.02; 0.07] 2.1% 1.9%
Song 2016 3 147 0.02 [0.00; 0.06] 1.0% 1.7%
Suo 2019 4 96 0.04 [0.01; 0.10] 0.6% 1.6%
Suo 2020 4 100 0.04 [0.01; 0.10] 0.6% 1.6%
ma 2017 0 56 0.00 [0.00; 0.06] 0.4% 1.4%
Song 2019 2 65 0.03 [0.00; 0.11] 0.4% 1.5%
Wang 2019 7 27 0.26 [0.11; 0.46] 0.2% 1.0%
Song 2020 19 650 0.03 [0.02; 0.05] 4.2% 2.0%
Cui 2021 13 506 0.03 [0.01; 0.04] 3.3% 1.9%
Tang 2020 9 117 0.08 [0.04; 0.14] 0.8% 1.7%
Common effect model 8865 0.03 [0.02; 0.03] 57.4% --
Random effects model 0.03 [0.02; 0.04] -- 50.4%
Heterogeneity: I^2 = 70%, τ^2 = 0.0022, p < 0.01
method = agar
Zhang 2002 1 164 0.01 [0.00; 0.03] 1.1% 1.8%
Ruan 2007 0 47 0.00 [0.00; 0.08] 0.3% 1.3%
Ruan 2007 0 54 0.00 [0.00; 0.07] 0.4% 1.4%
Ruan 2007 2 102 0.02 [0.00; 0.07] 0.7% 1.6%
Zheng 2010 0 77 0.00 [0.00; 0.05] 0.5% 1.5%
Xu 2012 0 120 0.00 [0.00; 0.03] 0.8% 1.7%
Zhang 2015 3 200 0.01 [0.00; 0.04] 1.3% 1.8%
Pan 2020 0 467 0.00 [0.00; 0.01] 3.0% 1.9%
Shen 2017 3 105 0.03 [0.01; 0.08] 0.7% 1.6%
Liu 2018 0 398 0.00 [0.00; 0.01] 2.6% 1.9%
Yang 2019 0 232 0.00 [0.00; 0.02] 1.5% 1.8%
Sun 2018 0 127 0.00 [0.00; 0.03] 0.8% 1.7%
Lv 2020 7 557 0.01 [0.01; 0.03] 3.6% 1.9%
Yu 2019 0 145 0.00 [0.00; 0.03] 0.9% 1.7%
Jiang 2021 22 1204 0.02 [0.01; 0.03] 7.8% 2.0%
Zhang 2021 0 231 0.00 [0.00; 0.02] 1.5% 1.8%
Xu 2020 0 100 0.00 [0.00; 0.04] 0.6% 1.6%
Luo 2020 2 207 0.01 [0.00; 0.03] 1.3% 1.8%
Common effect model 4537 0.00 [0.00; 0.01] 29.4% --
Random effects model 0.00 [0.00; 0.01] -- 30.9%
Heterogeneity: I^2 = 62%, τ^2 = 0.0017, p < 0.01
method = KB
Cao 2007 32 85 0.38 [0.27; 0.49] 0.6% 1.6%
Lin 2009 3 137 0.02 [0.00; 0.06] 0.9% 1.7%
Tan 2011 27 120 0.22 [0.15; 0.31] 0.8% 1.7%
Luo 2013 24 300 0.08 [0.05; 0.12] 1.9% 1.9%
Meng 2016 4 155 0.03 [0.01; 0.06] 1.0% 1.7%
Zhou 2018 3 150 0.02 [0.00; 0.06] 1.0% 1.7%
Zhang 2020 68 196 0.35 [0.28; 0.42] 1.3% 1.8%
Wang 2018 1 39 0.03 [0.00; 0.13] 0.3% 1.2%
Jiang 2020 14 553 0.03 [0.01; 0.04] 3.6% 1.9%
He 2021 20 200 0.10 [0.06; 0.15] 1.3% 1.8%
Common effect model 1935 0.08 [0.07; 0.09] 12.5% --
Random effects model 0.10 [0.04; 0.18] -- 17.0%
Heterogeneity: I^2 = 96%, τ^2 = 0.0331, p < 0.01
method = PCR
Gao 2020 6 111 0.05 [0.02; 0.11] 0.7% 1.6%
Common effect model 111 0.05 [0.02; 0.11] 0.7% --
Random effects model 0.05 [0.02; 0.11] -- 1.6%
Heterogeneity: not applicable
Common effect model 15448 0.02 [0.02; 0.03] 100.0% --
Random effects model 0.03 [0.02; 0.04] -- 100.0%
Heterogeneity: I^2 = 90%, τ^2 = 0.0094, p < 0.01
0 0.1 0.2 0.3 0.4

Figure S23

Tetracycline [by method]

Study	Events	Total	Proportion	95%-CI	Weight (common)	Weight (random)
**method = ETEST**						
Wang 2019	3	23	0.13	[0.03; 0.34]	0.2%	1.8%
Wang 2019	7	50	0.14	[0.06; 0.27]	0.5%	2.5%
Gao 2010	0	41	0.00	[0.00; 0.09]	0.4%	2.3%
Gao 2010	0	39	0.00	[0.00; 0.09]	0.4%	2.3%
Gao 2010	1	24	0.04	[0.00; 0.21]	0.2%	1.8%
Song 2014	21	600	0.04	[0.02; 0.05]	5.6%	3.8%
Zhang 2015	18	371	0.05	[0.03; 0.08]	3.5%	3.7%
Liu 2018	21	1117	0.02	[0.01; 0.03]	10.5%	3.9%
Liu 2019	18	804	0.02	[0.01; 0.04]	7.6%	3.8%
Zhang 2015	69	950	0.07	[0.06; 0.09]	8.9%	3.9%
Zhang 2015	51	700	0.07	[0.05; 0.09]	6.6%	3.8%
Song 2016	2	58	0.03	[0.00; 0.12]	0.6%	2.7%
Xie 2018	2	288	0.01	[0.00; 0.02]	2.7%	3.6%
Fu 2017	23	324	0.07	[0.05; 0.10]	3.1%	3.7%
Suo 2019	4	96	0.04	[0.01; 0.10]	0.9%	3.1%
Suo 2020	5	100	0.05	[0.02; 0.11]	0.9%	3.1%
Song 2019	4	65	0.06	[0.02; 0.15]	0.6%	2.8%
Wang 2019	5	27	0.19	[0.06; 0.38]	0.3%	1.9%
Song 2020	27	650	0.04	[0.03; 0.06]	6.1%	3.8%
Cui 2021	32	506	0.06	[0.04; 0.09]	4.8%	3.8%
Tang 2020	1	117	0.01	[0.00; 0.05]	1.1%	3.2%
Common effect model		6950	0.04	[0.03; 0.04]	65.5%	--
Random effects model			0.04	[0.03; 0.05]	--	65.2%
Heterogeneity: $I^2 = 83\%$, $\tau^2 = 0.0038$, $p < 0.01$						
**method = agar**						
Zheng 2010	0	77	0.00	[0.00; 0.05]	0.7%	2.9%
Shen 2017	1	105	0.01	[0.00; 0.05]	1.0%	3.1%
Liu 2018	0	398	0.00	[0.00; 0.01]	3.7%	3.7%
Yang 2019	0	232	0.00	[0.00; 0.02]	2.2%	3.5%
Sun 2018	0	127	0.00	[0.00; 0.03]	1.2%	3.2%
Lv 2020	0	557	0.00	[0.00; 0.01]	5.2%	3.8%
Jiang 2021	4	1204	0.00	[0.00; 0.01]	11.3%	3.9%
Common effect model		2700	0.00	[0.00; 0.00]	25.4%	--
Random effects model			0.00	[0.00; 0.00]	--	24.2%
Heterogeneity: $I^2 = 0\%$, $\tau^2 = 0$, $p = 0.48$						
**method = KB**						
Luo 2013	0	300	0.00	[0.00; 0.01]	2.8%	3.6%
Jiang 2020	4	553	0.01	[0.00; 0.02]	5.2%	3.8%
Common effect model		853	0.00	[0.00; 0.01]	8.0%	--
Random effects model			0.00	[0.00; 0.01]	--	7.4%
Heterogeneity: $I^2 = 66\%$, $\tau^2 = 0.0012$, $p = 0.09$						
**method = PCR**						
Gao 2020	14	111	0.13	[0.07; 0.20]	1.0%	3.2%
Common effect model		111	0.13	[0.07; 0.20]	1.0%	--
Random effects model			0.13	[0.07; 0.20]	--	3.2%
Heterogeneity: not applicable						
**Common effect model**		**10614**	**0.02**	**[0.02; 0.02]**	**100.0%**	**--**
**Random effects model**			**0.02**	**[0.01; 0.04]**	**--**	**100.0%**
Heterogeneity: $I^2 = 92\%$, $\tau^2 = 0.0087$, $p < 0.01$						

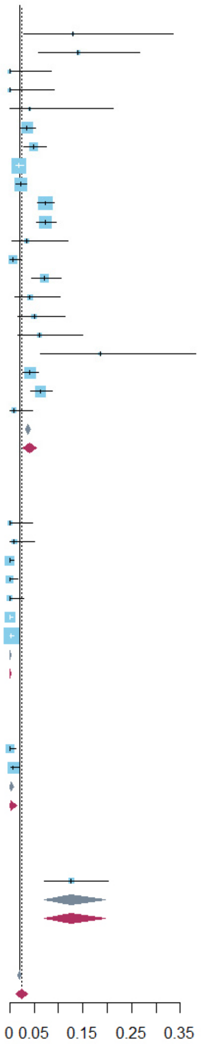

Figure S24

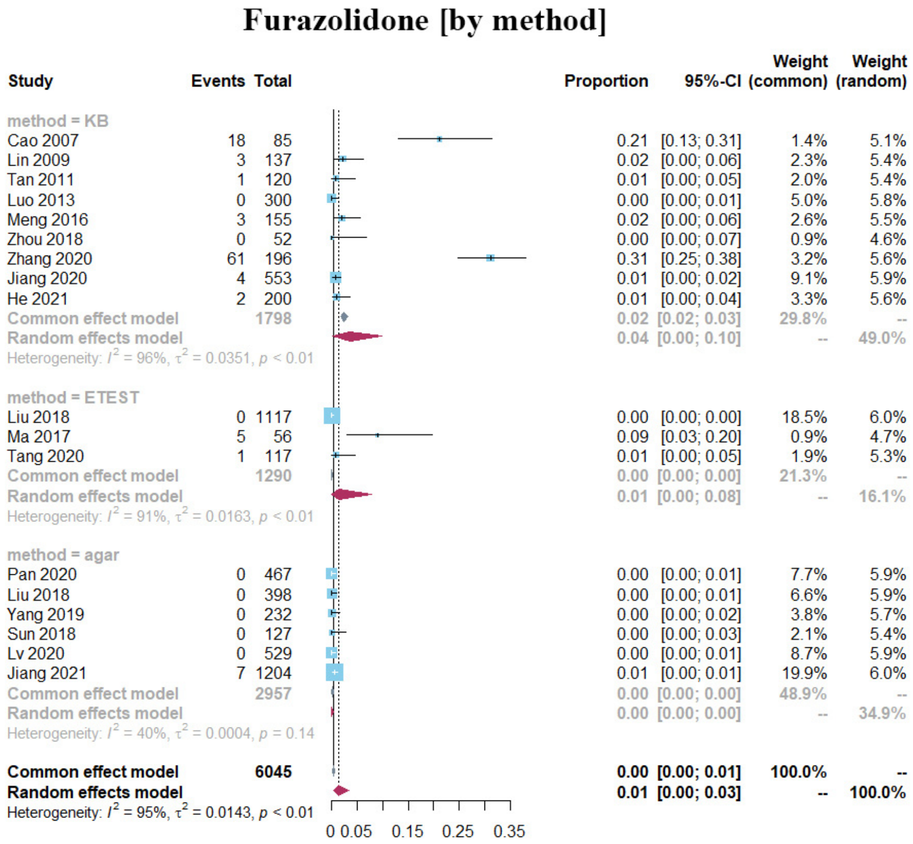

Furazolidone [by method]
Study Events Total Proportion 95%-CI Weight (common) Weight (random)
method = KB
Cao 2007 18 85 0.21 [0.13; 0.31] 1.4% 5.1%
Lin 2009 3 137 0.02 [0.00; 0.06] 2.3% 5.4%
Tan 2011 1 120 0.01 [0.00; 0.05] 2.0% 5.4%
Luo 2013 0 300 0.00 [0.00; 0.01] 5.0% 5.8%
Meng 2016 3 155 0.02 [0.00; 0.06] 2.6% 5.5%
Zhou 2018 0 52 0.00 [0.00; 0.07] 0.9% 4.6%
Zhang 2020 61 196 0.31 [0.25; 0.38] 3.2% 5.6%
Jiang 2020 4 553 0.01 [0.00; 0.02] 9.1% 5.9%
He 2021 2 200 0.01 [0.00; 0.04] 3.3% 5.6%
Common effect model 1798 0.02 [0.02; 0.03] 29.8% --
Random effects model 0.04 [0.00; 0.10] -- 49.0%
Heterogeneity: $I^2 = 96\%$, $\tau^2 = 0.0351$, $p < 0.01$
method = ETEST
Liu 2018 0 1117 0.00 [0.00; 0.00] 18.5% 6.0%
Ma 2017 5 56 0.09 [0.03; 0.20] 0.9% 4.7%
Tang 2020 1 117 0.01 [0.00; 0.05] 1.9% 5.3%
Common effect model 1290 0.00 [0.00; 0.00] 21.3% --
Random effects model 0.01 [0.00; 0.08] -- 16.1%
Heterogeneity: $I^2 = 91\%$, $\tau^2 = 0.0163$, $p < 0.01$
method = agar
Pan 2020 0 467 0.00 [0.00; 0.01] 7.7% 5.9%
Liu 2018 0 398 0.00 [0.00; 0.01] 6.6% 5.9%
Yang 2019 0 232 0.00 [0.00; 0.02] 3.8% 5.7%
Sun 2018 0 127 0.00 [0.00; 0.03] 2.1% 5.4%
Lv 2020 0 529 0.00 [0.00; 0.01] 8.7% 5.9%
Jiang 2021 7 1204 0.01 [0.00; 0.01] 19.9% 6.0%
Common effect model 2957 0.00 [0.00; 0.00] 48.9% --
Random effects model 0.00 [0.00; 0.00] -- 34.9%
Heterogeneity: $I^2 = 40\%$, $\tau^2 = 0.0004$, $p = 0.14$
Common effect model 6045 0.00 [0.00; 0.01] 100.0% --
Random effects model 0.01 [0.00; 0.03] -- 100.0%
Heterogeneity: $I^2 = 95\%$, $\tau^2 = 0.0143$, $p < 0.01$
0 0.05 0.15 0.25 0.35
